# ﻿New and noteworthy species of the genus *Epidendrum* (Orchidaceae, Laeliinae) from the Área de Conservación Privada La Pampa del Burro, Amazonas, Peru

**DOI:** 10.3897/phytokeys.227.101907

**Published:** 2023-06-01

**Authors:** Jessy Patricia Arista, Eric Hágsater, Elizabeth Santiago, José D. Edquén, Elí Pariente, Manuel Oliva, Gerardo A. Salazar

**Affiliations:** 1 Instituto de Investigación, Innovación y Desarrollo del Sector Agrario y Agroindustrial (IIDAA), Universidad Nacional Toribio Rodríguez de Mendoza de Amazonas, Chachapoyas, Amazonas, Peru; 2 Escuela de Posgrado, Universidad Nacional Toribio Rodríguez de Mendoza de Amazonas, Chachapoyas, Amazonas, Peru; 3 Herbario AMO, Montañas Calizas 490, Lomas de Chapultepec. Miguel Hidalgo, Mexico City, 11000, Mexico; 4 Instituto de Investigación para el Desarrollo Sustentable de Ceja de Selva (INDES–CES), Universidad Nacional Toribio Rodríguez de Mendoza de Amazonas, Chachapoyas, Amazonas, Peru; 5 Departamento de Botánica, Instituto de Biología, Universidad Nacional Autónoma de México, Mexico City, Mexico

**Keywords:** Dwarf forest on white sand, eastern Andean ridge, Lankester Composite Dissection Plates, moist or wet montane forest, range extension

## Abstract

Fourteen species of the genus *Epidendrum*, recently collected in the Área de Conservación Privada La Pampa del Burro (ACPPB), five of them new to science (*Epidendrumechinatiantherum***sp. nov.**, *E.imazaense***sp. nov.**, *E.parvireflexilobum***sp. nov.**, *E.rosulatum***sp. nov.**, and *E.ochrostachyum***sp. nov.**), are described and illustrated. The other species include a new record for Peru (*E.acrobatesii*) and four for the department of Amazonas (*E.brachyblastum*, *E.forcipatum*, *E.mavrodactylon*, and *E.tridens*). *Epidendrumenantilobum* is here considered a synonym of *Epidendrumbrachyblastum*. The type locality of *Epidendrumcryptorhachis*, originally stated as Ecuador, Guayabamba, is corrected to indicate that it refers to the valley of Guayabamba, Rodríguez de Mendoza, Amazonas, Peru. Our results show the need to continue conducting botanical exploration in the ACPPB as a baseline for subsequent studies, including a full inventory of the orchid diversity.

## ﻿Introduction

*Epidendrum* L. is the most species-rich Neotropical genus of Orchidaceae, with an estimated 2400 species, including undescribed species for which specimens or photographs have been recorded (e.g., Hágsater et al. 2016, 2019). Although earlier publications estimated the number of species at around 1500 (Hágsater and Soto Arenas 2005), the number of species presently monographed and confirmed is approximately 1900, with many added every year. The International Plant Names Index (IPNI, https://ipni.org/; accessed 12 March 2023) presently includes some 3425 names under *Epidendrum*, but many of these have been transferred to other genera, others are considered synonyms, and ten are subgeneric names. The genus is distributed from the southern United States to northern Argentina, and its species occupy nearly every habitat suitable for plant life, including mangroves and coastal dunes, tropical wet to seasonally dry forests, various types of montane forest and scrub, and Andean paramos, ranging from sea level to 4200 m elevation (Hágsater and Soto Arenas 2005; Hágsater and Krahl 2020). The plants are epiphytic, terrestrial, or lithophytic, displaying caespitose, repent, or pendulous growth forms. The stems often are slender and cane-like, but in some species the stems are thickened into pseudobulbs; and the leaves are usually distichously arranged along the stems. The flowers display a wide variety of sizes, colors, and fragrances, but the common theme among them is that the base of the labellum is united throughout the column length to form a narrow tunnel leading to a cuniculus that penetrates the pedicellate ovary to a varying extent. The small number of published observations indicate that pollination by diurnal and nocturnal lepidopterans is common, with some instances of hummingbird pollination (Dressler 1961, Hágsater and Soto Arenas 2005, Silveira et al. 2023).

According to a recent compilation, the orchid flora of Peru includes about 2900 species and 204 genera (Goicochea et al. 2019), in contrast with a conservative estimate given by Peru’s Ministerio del Ambiente (MINAM), in which only 2206 species were recorded (MINAM 2015). Many species are considered endemic to the country and endemism seems to be concentrated in the departments of Amazonas, Apurímac, Ayacucho, Cajamarca, Cusco, Junín, Puno, and San Martín (Roque and León 2006). *Epidendrum* stands out as the largest genus of Orchidaceae in Peru, with about 480 species recorded (Quispe-Melgar et al. 2022).

In this study, we present initial results of our ongoing project aimed at documenting the orchid diversity present in the Área de Conservación Privada La Pampa del Burro. The information available on the floristic diversity of the area, and particularly that of the Orchidaceae, is very limited, with only 12 species of this family recorded to date (Shanee et al. 2012). Here, we focus on five undescribed and nine little-known species of the genus *Epidendrum* recorded in the ACPPB. Every species is described and illustrated with a detailed color plate, and information on their distribution and habitat, and a comparison with similar species, is provided. We aim to contribute to the knowledge of the genus *Epidendrum* in Peru and, particularly, to the orchid inventory of a highly diverse yet botanically unexplored protected area, as a baseline for subsequent studies. This contribution is not a complete inventory of the many species of *Epidendrum* in the ACPPB, but rather a first installment dealing with some of the new species found and new records for Peru or the region.

## ﻿Materials and methods

### ﻿Study area

**Figure 1.**
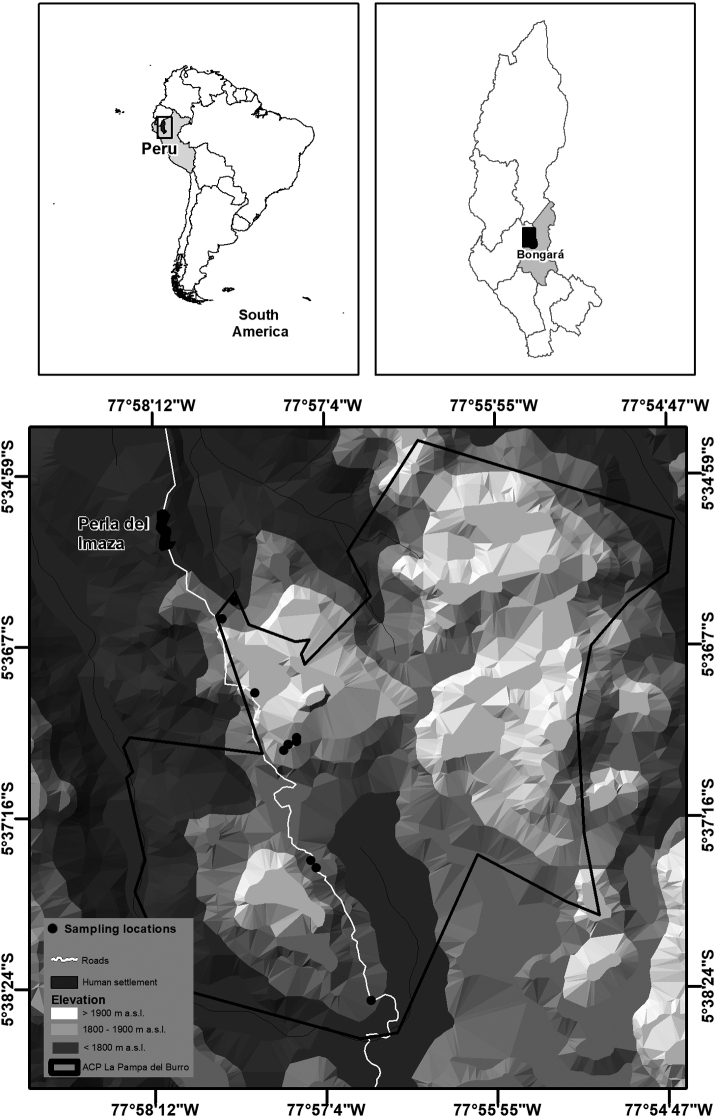
Location of the Área de Conservación Privada La Pampa del Burro in the province of Bongará, department of Amazonas, Peru.

**Figure 2.**
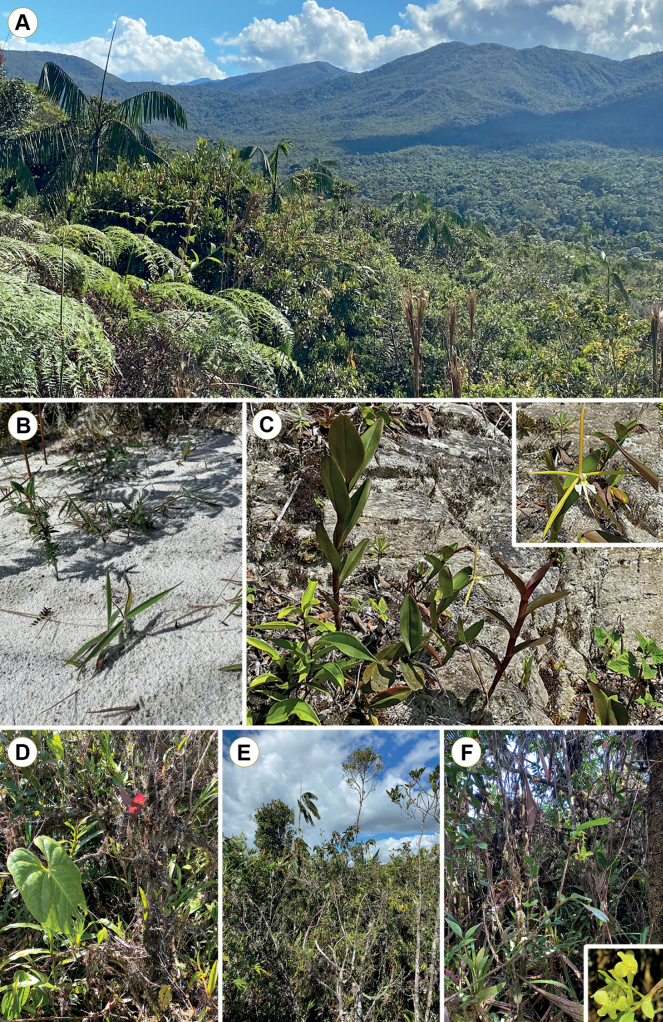
Overview of the vegetation in the Área de Conservación Privada La Pampa del Burro **A** forested low hills and slopes, with dwarf forest on white sand in the forefront **B** exposed white sand in a cleared area **C***Epidendrumtridens* on white sand on a roadside embankment; inset: close-up of a flower **D** dense tangle of epiphytes and climbers in the understory of the dwarf forest **E** canopy of the dwarf forest **F***Epidendrumweigendii*, a climber orchid; inset: close-up of an inflorescence (Photographs by G. A. Salazar).

The Área de Conservación Privada La Pampa del Burro (ACPPB) belongs to the settlement of Perla del Imaza of the Comunidad Campesina de Yambrasbamba, province of Bongará, department of Amazonas, northeastern Peru, covering a surface of 2,776.96 ha on the eastern Andean range at 1750–1900 m (ca. 5°34’59"–5°38’24"S, 77°54’47"–77°58’12"W; Fig. 1). The ACPPB forms a natural biological corridor between five other conservation units, namely the Bosque de Protección Alto Mayo, the Reserva Comunal Chayu Nain, the Área de Conservación Privada Abra Patricia, the Zona Reservada Río Nieva, and the Santuario Nacional Cordillera de Colán. Hence, the ACPPB is a key area to maintain the connectivity between various ecosystems and the continuity of natural cycles (Shanee et al. 2012). Two main ecosystems are present in the ACPPB, dwarf forest on white sand and moist or wet montane forest. The dwarf forest on white sand is a very particular type of vegetation characterized by high endemism (Ruokolainen and Tuomisto 1998) due to the nutrient deficiency and high acidity of the quartzite sand, which are highly restrictive for species not specialized to these conditions (Neill et al. 2014; Fig. 2). In contrast, the moist or wet montane forest is one of the most diverse ecosystems in the world as far as the number of species is concerned (e.g., Krömer et al. 2007). Moreover, the ACPPB was proposed as an important conservation area to preserve the habitat of the yellow-tailed woolly monkey, *Lagothrixflavicauda* Humboldt, 1812, the Peruvian night monkey, *Aotusmiconax* Thomas, 1927, and the long-whiskered owlet, *Xenoglauxloweryi* O’Neill & Graves, 1977.

### ﻿Field collections and herbarium taxonomy

Material of the species reported here was collected during two field trips conducted in August 2021 and July 2022. A permit for scientific research was granted by Peru’s Servicio Nacional Forestal y de Fauna Silvestre (SERFOR), with authorization code No. AUT-IFL-2021-033. Each species was photographed in the field and the laboratory using a digital camera (Nikon D850, Nikon Corporation, Tokyo, Japan) equipped with a Nikkor 60 mm, 2.8 lens and a Nikon Speedlight SB–70 (both from Nikon). Lankester Composite Dissection Plates (LCDP; Karremans 2020) were prepared with ADOBE PHOTOSHOP v. 24.1.0. Specimens of each species were pressed, dried, and deposited in the herbarium of the Universidad Nacional Toribio Rodríguez de Mendoza, Chachapoyas, Peru (KUELAP).

For the taxonomic identification of the various species, each specimen was first assigned to the appropriate group within the genus *Epidendrum* according to E. Hágsater and collaborators (Hágsater 1985), and subsequently it was tentatively identified to species level within the group. With a first tentative identification, a search was made for published illustrations, plates and descriptions in the various series of The Genus *Epidendrum* in Icones Orchidacearum (Hágsater and Salazar 1993; Hágsater et al. 1999; Hágsater and Sánchez Saldaña 2001, 2004, 2006, 2007, 2008, 2009, 2010, 2013, 2015, 2016a, b; Hágsater and Santiago 2018, 2019, 2020a, b, 2021, 2022a, b), Icones Orchidacearum Peruviarum (Bennett and Christenson 1993, 1995a, b, 1998, 2001), and Icones Plantarum Tropicarum (Dodson and Dodson 1980a, b, c, 1982, 1984, 1989a, b; Vásquez and Dodson 1982; Dodson and Bennett 1989a, b). In addition, searches were carried out in AMODATA, the database of the AMO Herbarium (Hágsater and Sánchez Saldaña 2016b), which includes some 150000 digital color images taken in the field, as well as of herbarium specimens photographed since 1976 in herbaria in the United States, Europe and tropical America by the staff of AMO, in search of specimens under the preliminary name or from the relevant geographic region, to identify possible specimens of the same entity. The herbaria for which *Epidendrum* material was available for comparison include AAU, AMES, AMO, ANDES, B (photographs taken before its destruction), BM, BR, BRIT, C, CAS. CHAX, CM, COL, CPUN, CTES, CUVC, CUZ, E, ENCB, F, FI, G, GH, GOET, HAO, HB, HBG, HCEN, HNOP, HOXA, HURP, HUSA, HUT, INPA, ITA, JAUM, K, KUELAP, L, LE, LL, M, MA, MEXU, MICH, MO, MOL, NY, OXF, P, PR, PRC, PRG, QCA, QCNE, R, RENZ, S, SEL, TEX, TNS, U, UC, UFV, US, USF, USM, VEN, W, WIS, and WRSL. Most specimens were positively identified as a published species. The material found in the abovementioned sources was studied, in many instances allowing to widen previously known distribution ranges. In some cases, additional specimens were found to have been recorded earlier but filed without an identification or under the wrong name.

The information provided in the following for each species includes a full description, a LCDP color figure and, for narrowly distributed species with a small number of records, we cite all Peruvian material found. Additionally, in some instances specimens from neighboring Ecuador are also listed, not only when the type corresponds to that country, but in the few cases where the known range includes southern Ecuador, which shares the same biomes. In the case of common species widely distributed throughout the Andes, only Peruvian materials are cited. Specimens refer to herbarium sheets or in some cases flowers in spirit. Other Records refer to illustrations or color digital images where no herbarium specimens were prepared or found. Reference is made to where these materials are kept. Many have been gathered from Facebook, GBIF, or personal correspondence.

Finally, as this is not a floristic treatment, no key to the species of the genus for the area is given.

## ﻿Taxonomic treatment

### ﻿ Epidendrum acrobatesii

Taxon classificationPlantaeAsparagalesOrchidaceae

Hágsater & Dodson, Icon. Orchid. 4: t. 402. 2001.

6CC1E448-BB5E-53B5-9235-73F3DCA98130

Fig. 3

#### Type material.

**Ecuador. Loja**: N slope of Nudo de Sabanilla S of Yangana on road to Valladolid, 4°28'S, 79°10'W, 2500 m, 24 Feb. 1988, *U. Molau & B. Eriksen 3191* (holotype: GB!; isotypes: AAU! QCA!).

#### Description.

Epiphytic or terrestrial, monopodial, branching, erect ***herb***, 31–34 cm tall including inflorescence. ***Roots*** 1–3 mm in diameter, emerging only from base of primary stem, fleshy, thick. ***Stems*** cane-like, erect, somewhat sinuous, branching from sub-apical internodes; primary stem 16 × 0.5 cm; secondary stems 1.5–14.6 × 0.3–0.4 cm. ***Leaves*** ca. 19 on primary stem, 5–14 on secondary stems, distributed throughout stems, usually only apical 3–5 leaves remaining at flowering, alternate, articulate; sheath 0.7–1.4 × 0.3–0.5 cm, tubular, rugose, striated; blade 2.2–3.8 × 0.7–1.4 cm, length:width 3:1, apical leaf usually reduced, elliptic, mucronate, light green on both sides, apical margin minutely erose-dentate, spreading. ***Spathe*** lacking. ***Inflorescence*** up to 7.6 cm long including flowers, apical, flowering only once, racemose (sometimes with a short branch near base), erect in early stages, becoming arching-nutant as it develops; peduncle 5–7 mm long, terete, rachis 15–33 mm long, developing as new flowers are formed, flexuous, compact, ornamented with a short keel at base of each floral bract. ***Floral bracts*** 1–3 mm long, much shorter than ovary, ovate, conduplicate, acute to obtuse. ***Flowers*** 6–12, successive, 1–2 open at a time in different stages, with smaller buds present, erect, facing upwards, yellow to green; apparently not fragrant. ***Sepals*** 10–12 × 3.5–4.2 mm, free, spreading, narrowly elliptic to oblanceolate, acute, slightly aristate, especially the lateral sepals, 5-veined, margins entire, revolute. ***Petals*** 12 × 0.6–1.8 mm, free, spreading, linear or linear-obcuneate, obtuse to rounded, 1-veined, margins entire, spreading. ***Lip*** 5.0–7.7 × 7.0–9.0 mm, cordiform in general outline, deeply 3-lobed, somewhat concave basally in natural position with lateral margins and apex more or less revolute; bicallose, calli 3.7 × 0.6 mm, elongate, parallel, with a mid-rib running to apical sinus, disc with multiple thickened veins, converging basally and radiating apically; lateral lobes 3.2 × 7.0 mm, semi-sagittate, apex acute, margins progressively short-laciniate; mid-lobe 3.1 × 3.2 mm, quadrate to obcuneate, apex truncate to emarginate, with a wide sinus, margins entire to erose. ***Column*** 7–8 mm long, thin, straight. ***Clinandrium hood*** truncate, margin entire. ***Anther*** reniform. 4-celled. ***Pollinia*** 4, obovate, laterally compressed, inner face of each pair flat; caudicles short. ***Rostellum*** apical, slit; viscarium semi­liquid, transparent. ***Cuniculus*** penetrating half of pedicellate ovary and widened toward the middle of the ovary, forming a narrowly ellipsoid vesicle. ***Ovary*** 17–24 × 1.6–3.2 mm, slightly inflated ventrally beyond middle forming an elongate vesicle, but narrow near apex, furrowed. ***Capsule*** not seen.

**Figure 3.**
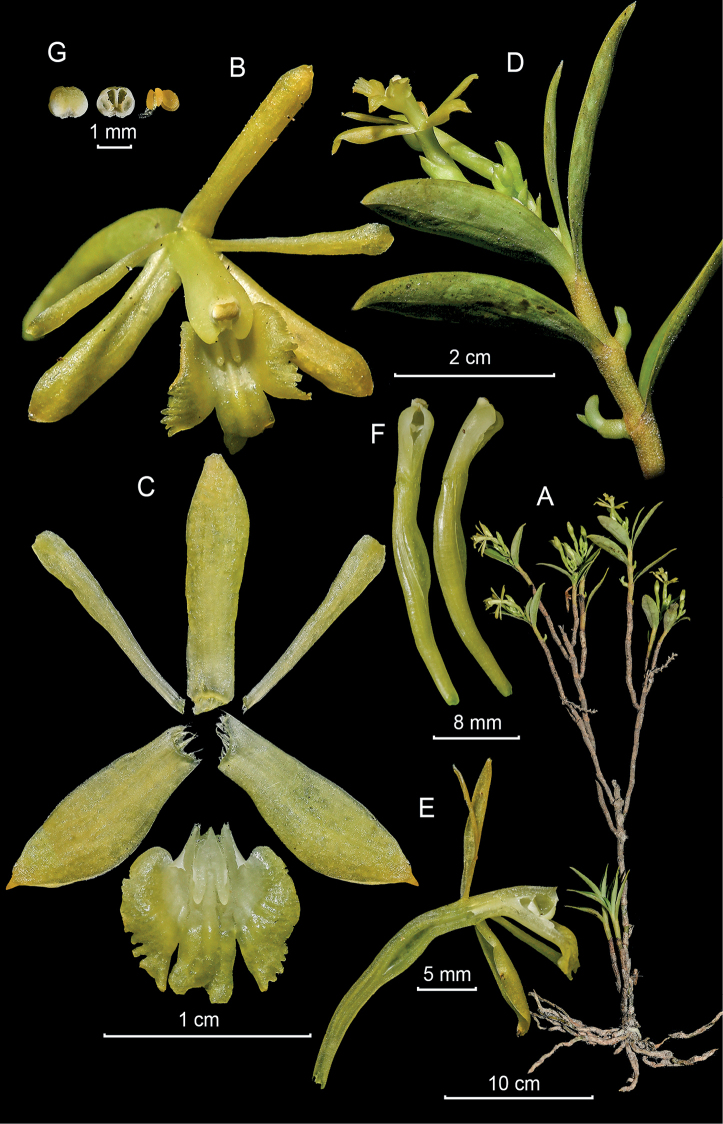
*Epidendrumacrobatesii* from *Arista et al. 21***A** habit **B** flower **C** dissected perianth **D** inflorescence at apex of stem **E** longitudinal section of flower **F** column and ovary-pedicel from below (left) and side (right) **G** anther from above (left) and below (right), and pollinarium (Photographs by J. D. Edquén; plate prepared by A. Cisneros).

#### Additional specimens examined.

**Peru. Amazonas**: La Pampa del Burro, 22 Aug. 2021, *Arista et al. 21* (KUELAP!); **Pasco**: Oxapampa, Parque Nacional Yanachaga–Chemillén, parte alta de la trocha Tunqui–Cajonpata, sector Tunqui, 1950 m, 31 Oct. 2007, *Monteagudo 15793* (HOXA!).

#### Other records.

**Peru. Amazonas**: Chachapoyas: Distr. Longuita, Fortaleza Kuelap, 14 Nov. 2019, *Harding s.n.*, digital images (AMO!); Bongará: Yambrasbamba; Progreso, 30 Dec. 2019, *Velásquez s.n.*, digital images (AMO!); **San Martín**: Prov. Rioja, Distr. Pardo Miguel Naranjos, 1962 m, 6 Nov. 2015, *Edquén 2042*, digital images (AMO!); *Ibid. loc.*, 1691 m, 22 Feb. 2017, *Edquén 2043*, digital images (AMO!); *Ibid. loc.*, 1790 m, 20 Feb. 2017, *Edquén 2044*, digital images (AMO!); hort. Moyobamba, 9 Jan. 2017, *Goicochea s.n.*, digital images (AMO!).

#### Distribution.

Widespread from Ecuador (Napo and Zamora–Chinchipe) south to Oxapampa, Pasco, in central Peru. The range of the species along the upper eastern slope of the Andes in Ecuador and central Peru spans some 1150 km, with at least 7 known localities. Growing at 1691–2500 m.

#### Habitat and ecology.

Epiphytic, found on very wet fallen logs with abundant moss, in primary montane humid forest or elfin forest, often with afternoon fog. Sometimes at the base of *Chusquea* Kunth. Also terrestrial on edge of roadside banks of white sand with abundant organic material in elfin forest.

#### Phenology.

Flowering throughout the year.

#### Taxonomic notes.

*Epidendrumacrobatesii* has leaves with a length:width proportion about 3:1, elliptic, with the margin minutely erose–dentate, the inflorescence 5 cm long, with 6–12 greenish yellow flowers, sepals 10–12 mm long, petals linear to linear–obcuneate, and the lateral lobes of the lip nearly as long as the mid-lobe, which is apically truncate to emarginate. *Epidendrumbatesii* Dodson has proportionately narrower leaves, 2.5–3.4 × 0.6–0.8 cm, length:width 4:1, the mid-lobe of the lip shorter and entire, 2.3–2.5 mm long. *Epidendrumoxybatesii* Hágsater & Dodson from northern Ecuador is distinguished by the long, acicular mid-lobe of the lip.

This is an addition to the flora of Peru. The Batesii Group has a peculiar architecture in that the new stems produced from the apical half of the basal stem can be as long as the primary stem, and those closest to the apex are produced first, with shorter stems being later produced further down the primary stem.

### ﻿ Epidendrum brachyblastum

Taxon classificationPlantaeAsparagalesOrchidaceae

Hágsater & Dodson, Icon. Orchid. 7: t. 713. 2004.

C6B0A75A-2F48-5B87-B265-9EA3B6EF88D1

Fig. 4

#### Type material.

**Ecuador. Pastaza**: Mera, 11 km, cañada del Río Anzú, 1225 m, collected 10 Dec. 1986, flowered in cultivation 6 May 1987, *E. Hágsater & C. H. Dodson 9093* (holotype: AMO, spirit! flower card and color slides, AMO!).

#### New taxonomic synonym.

*Epidendrumenantilobum* Hágsater, Icon. Orchid. 16(1): pl. 1616 (2018) syn. nov. Type: Peru. San Martín: Rioja–Pomacochas road, below Venceremos, ca 20 km NW of Rioja, near Restaurante El Amigo, 1600 m, 8 Feb. 1984, *A. H. Gentry & D. N. Smith 45148* (holotype: NY!; isotype: MO-349072!)

#### Description.

Epiphytic, sympodial, caespitose, erect ***herb***, 30–55 cm tall including inflorescence. ***Roots*** 2 mm in diameter, basal, fleshy. ***Stems*** 6–53 × 0.3–0.5 cm, simple, cane-like, terete, thin, straight. ***Leaves*** 5–12 distributed along apical 2/3 of stem, sub-erect, alternate, sub-coriaceous; sheath 10–14 mm long, tubular, minutely striated; blade 3.3–11.5 × 1.0–2.5 cm, narrowly elliptic to oblong-elliptic, acuminate, minutely apiculate; margin entire, spreading. ***Spathe*** lacking. ***Inflorescence*** 5–12.5 cm long, apical, racemose to paniculate, compact, arched; peduncle 4 cm long, short, terete, thin, nearly totally covered by 1–3 basal bracts, 1.5–2.2 cm long, terete, triangular-lanceolate, acuminate, embracing; rachis 2–3.5 cm long, short, terete, thin, straight to arching. ***Floral bracts*** 4–14 mm long, progressively shorter, triangular-lanceolate, acuminate. ***Flowers*** 9–25 per raceme, simultaneous, resupinate, lip always oriented toward rachis, medium to dark green, lip and apical half of column white; fragrance not registered. ***Sepals*** 6.0–7.0 × 3.2–3.8 mm, free, fleshy, spreading, slightly concave, obovate, obtuse, minutely apiculate, margins entire, spreading; dorsal sepal 3-veined; lateral sepals 3-veined, with lateral veins bifurcate from base and appearing 5-veined. ***Petals*** 5.0–6.1 × 1.0–1.6 mm, free, spreading, narrowly spatulate, obtuse, 1-veined, apical margin slightly erose, spreading. ***Lip*** 4.5–6.4 × 5.0–7.0 mm, united to column, 3-lobed, base cordate; bicallose, calli thin, short, disc provided with a low, rounded mid-rib reaching apical sinus; lateral lobes 2.4–2.8 × 1.3–3.0 mm, falcate, narrow, sub-acute, posterior margin somewhat erose; mid-lobe 2.0–3.7 × 1.5–4.5 mm, isthmus sub-rectangular, gradually narrower then deeply bifid toward the apex, lobes long, narrowly triangular, cirrhose, acuminate, divaricate, apices revolute. ***Column*** 5–8 mm long, slightly arched, base thin, abruptly thickened ventrally, with a pair of truncate lateral wings. ***Rostellum*** sub-apical, slit; viscarium semi-liquid. ***Lateral lobes of stigma*** half as long as stigmatic cavity. ***Anther*** ovoid, 4-celled. ***Pollinia*** 4, bird-wing-type, unequal, inner pair about ¾ size of outer pair. ***Cuniculus*** without penetrating pedicellate ovary, narrow at base and widening toward entrance, unornamented. ***Ovary*** 7–15 mm long including pedicel, terete, thin, not inflated, unornamented, furrowed. ***Capsule*** not seen.

**Figure 4.**
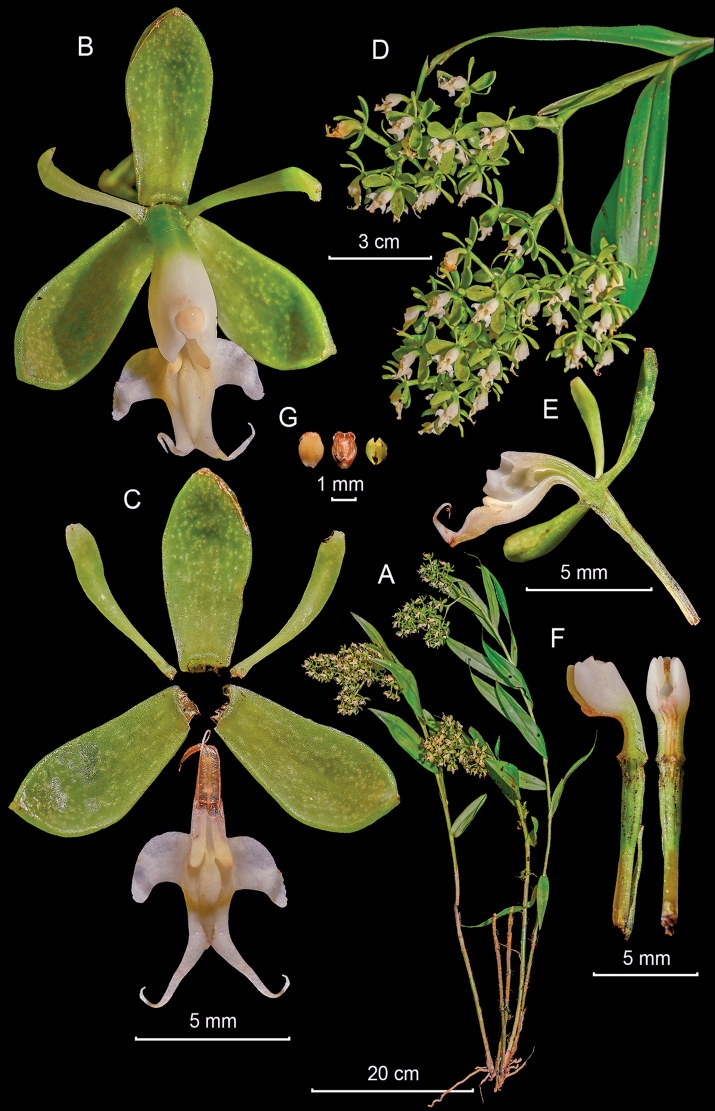
*Epidendrumbrachyblastum* from *Arista et al. 148***A** habit **B** flower **C** dissected perianth **D** inflorescence at apex of stem **E** longitudinal section of flower **F** column and ovary-pedicel from side (left) and below (right) **G** anther from above (left) and below (right), and pollinarium (Photographs by J. D. Edquén; plate prepared by A. Cisneros).

#### Additional specimens examined.

**Peru. Amazonas**: Bongará, Florida, Laguna Pomacocha, km 335 on the road to Rioja, 2360 m, 26 Jan. 1964, *Hutchinson 3809* (UC! USM!); Yambrasbamba, Perla del Imaza, Área de Conservación privada La Pampa del Burro, 1682 m, 28 Aug. 2021, *Arista et al. 148* (KUELAP!); **San Martín**: Carretera Rioja–Pedro Ruiz, 1450 m, 24 Mar. 1998, *van der Werff 15568* (MO!).

#### Other records.

**Peru. Amazonas**: alrededores de Chachapoyas, 2400 m, *Morón s.n.*, digital images (AMO!); **San Martín**: Moyobamba, Soritor, San José El Doncel, 3 Feb. 2021, *Bazan 1*, digital images (AMO!).

#### Distribution.

Presently known from Ecuador, near Mera in Pastaza, to northeastern Peru, in the border between Amazonas and San Martín, on the eastern Andean range near Moyobamba. The species has only been collected along two well-paved roads: the Troncal Amazónica, in Ecuador, and the Carretera Marginal de la Selva Fernando Belaunde Terry, in Peru. Growing at 1100–2360 m.

#### Habitat and ecology.

Epiphytic in wet forest.

#### Phenology.

Flowering from January to August.

#### Taxonomic notes.

*Epidendrumbrachyblastum* belongs to the Bicirrhatum Group, which is characterized by the caespitose habit, simple, cane-like stems, short, arching, pluriracemose, sub-capitate inflorescence, narrowly spathulate petals, 3-lobed lip, generally with cirrate apical lobes and unequal, laterally compressed pollinia, with the inner pair smaller. *Epidendrumbrachyblastum* medium to deep green flowers with the lip and apical half of the column snow white, the lateral lobes of the lip are narrow and falcate, the mid-lobe is split into two apical cirrhose lobes. It closely resembles *Epidendrumtiwinzaense* Hágsater & Dodson, which has pale green (vs. deep green) flowers, with the column and lip cream-colored, the lip with very wide dolabriform lateral lobes. It also closely resembles *E.bicirrhatum* D.E.Benn. & Christenson, which has opaque, pale cream-orange flowers, the sepals and petals green at the base, the tips of the calli keels lavender, and the column white at the base, cream-yellow above.

The type specimen of *E.brachyblastum* was prepared from a cultivated plant not in the best cultural conditions, with few, large flowers, and shorter apical lobes of the lip, thus not being a good representative of the species, which was later described as *E.enantilabium* Hágsater, here included in the synonymy of *E.brachyblastum*. *Hutchison 3809* was collected in 1964, but only recently identified as *E.enantilabium*, ergo the species was known earlier from Peru.

### ﻿ Epidendrum cryptorhachis

Taxon classificationPlantaeAsparagalesOrchidaceae

Hágsater, Icon. Orchid. 8: t. 823. 2006.

01F22E9E-E35E-540E-8470-784770C8E689

Fig. 5

#### Type material.

**Peru.** [**Amazonas**: Rodríguez de Mendoza: Valle de] Guayabamba, 8 Mar. 1877, *M. Vidal*-*Sénège s.n.* (holotype: P!; isotype: P!). [Locality corrected, see note under Distribution.]

#### Description.

Lithophytic or epiphytic, sympodial, caespitose, erect and arching ***herb***, 17–43 cm tall including inflorescence. ***Roots*** ca. 2–3 mm in diameter, basal, scarce, thin. ***Stems*** 6–11 × 0.2–0.6 cm, simple, short, cane-like, terete at base, laterally compressed toward apex, thin, flexuous. ***Leaves*** 3–6, distributed throughout stems, alternate, sub-erect; sheath 0.8–2.0 × 0.2–0.6 cm, infundibuliform when dry, minutely striated; blade 2.8–10 × 0.4–0.8 cm, linear-lanceolate, acuminate, succulent and coriaceous, canaliculate, margin entire. ***Inflorescence*** 16–32 cm long, apical, paniculate, arching–nutant, densely, many-flowered; peduncle 6–16 × 0.07–0.4 cm, elongate, longer than leaves, thin, ancipitose, two-winged, slightly sinuous, provided with 1–5 bracts 1.7–3.2 × 0.1–0.4 cm, each subtending a raceme, tubular and ancipitose at base, conduplicate above, long, acuminate; panicle with 6–9 short, straight, short racemes 1.4–2.7 cm long, densely 8–10-flowered, sub-parallel to axis of inflorescence, enveloped at base by large conduplicate bracts 1.0–2.5 × 0.1–0.3 mm, similar to those of peduncle but progressively shorter toward apex. ***Floral bracts*** 1–2 mm long, small, shorter than ovary, triangular, acute. ***Flowers*** ca. 90, 4–14 per raceme, small, membranaceous, simultaneous, resupinate, sepals ochre to pale green, petals and lip yellow, column yellow tinged brown, anther brown red; fragrance not registered. ***Sepals*** spreading, free, 3-veined, margin entire, revolute; dorsal sepal 3.3–3.7 × 1.6–2.0 mm, ovate-elliptic, sub-acute, lateral sepals 3.5–4.2 × 1.0–2.2 mm, oblong, apex obtuse, slightly oblique, short apiculate. ***Petals*** 3.0–3.7 × 0.6–0.7 mm, free, parallel to column and embracing it, narrowly obovate, apex rounded, 1-veined, margin entire, revolute. ***Lip*** 3 × 5.3 mm, united to column, bilobed, widely hexagonal, base cordate, apex emarginate, margin entire, sides revolute in natural position, somewhat apron shaped; lobes 2.0–2.8 × 3.3–4.3 mm, from semi-orbicular to obliquely triangular-quadrate; bicallose, calli prominent, finger-like, short, sub-erect at apex, disc with a low-wide, mid-rib reaching apical sinus, and 3 low rounded thickenings on each side. ***Column*** 2.8 × 0.9 mm, short, slightly arching, apex oblique, with short, obliquely truncate fleshy column wings. ***Clinandrium hood*** short, concave, margin entire. ***Anther*** spherical, with a very low rounded keel in front, 4-celled. ***Pollinia*** 4, nearly lentil-shaped, laterally compressed, translucent; caudicles very short. ***Rostellum*** apical, slit; viscarium semi-liquid, transparent. ***Lateral lobes of stigma*** 1.7 mm long, very short, ¼ length of stigmatic cavity, very slender. ***Cuniculus*** penetrating 1/3 of ovary, much inflated behind perianth, smooth. ***Ovary*** 5.3 × 2 mm at apex (including vesicle), terete, glabrous, furrowed, thin along basal 2/3, ventrally inflated toward apical 1/3, forming a prominent globose vesicle. ***Capsule*** not seen.

**Figure 5.**
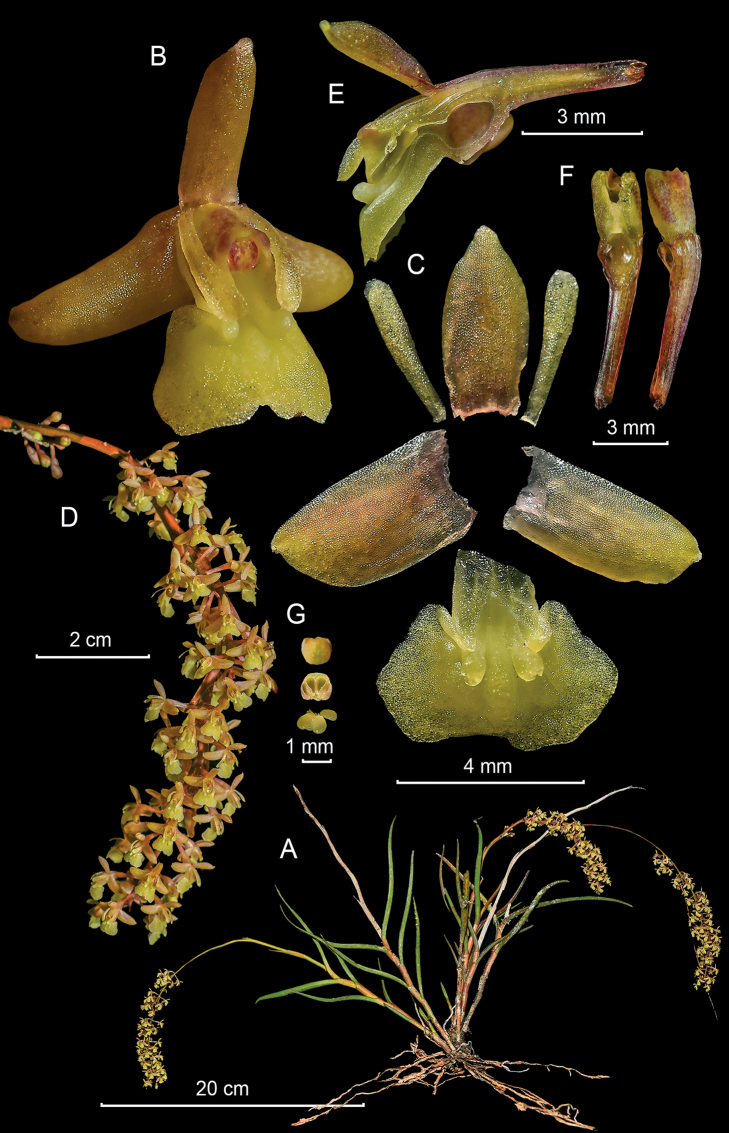
*Epidendrumcryptorhachis* from *Arista et al. 128***A** habit **B** flower **C** dissected perianth **D** inflorescence **E** longitudinal section of flower **F** column and ovary-pedicel from below (left) and side (right) **G** anther from above (up) and below (down), and pollinarium (Photographs by J. D. Edquén; plate prepared by A. Cisneros).

#### Additional specimens examined.

**Ecuador. Zamora-Chinchipe**: km 44 Loja-Zamora, *Dodson s.n. ex Missouri Bot. Garden* “*61-150-57* SEL! (illustration AMO!);” **Peru. Amazonas**: Bongará: Yambrasbamba, Perla del Imaza, Área de conservación privada La Pampa del Burro, 1763 m, 28 Aug. 2021, *Arista et al. 128* (KUELAP!); **Cajamarca**: San Ignacio, San José de Lourdes, Buenos Aires, 1880 m, 3 Nov. 2000, *Calatayud 804* (CUZ!); **Huánuco**: Between Huánuco and Pampayacu, 28 Jan. 1927, *Kanehira 26* (AMES!).

#### Other records.

**Peru. Amazonas**: Chachapoyas: Molinopampa, 2400–2700 m, 13 Nov. 2010, *Dalström 3240*, digital images (AMO!).

#### Distribution.

The species is presently known from the Amazon slope of the Andes in northern Peru and southern Ecuador, spanning some 720 km, at about 1750–2700 m elevation. Six localities have been identified, but the species is probably more widespread and common, and the terrain in between has not been thoroughly botanized. When first published, this species was thought to have come from Guayabamba in Ecuador, but recently we have learned that the Valley of Guayabamba is the valley of Rodriguez de Mendoza, south of Chachapoyas, in the department of Amazonas, Peru. Thus, this is the first confirmed record for Peru, though it had been collected earlier.

#### Habitat and ecology.

Lithophytic in cliffs and trunk epiphyte on *Inga* Mill. in humid premontane forest, “Ceja de Selva”, with *Cinchona* L. (Rubiaceae) and *Cedrela* P.Browne (Meliaceae).

#### Phenology.

Flowering from August to March.

#### Taxonomic notes.

*Epidendrumcryptorhachis* belongs to the Epidendropsis Group, Gracillimum Subgroup, characterized by the sympodial, caespitose, relatively small plants with non-thickened stems, long, paniculate inflorescences, delicate small flowers, and long, narrow, sub-coriaceous leaves. The species is recognized by long, many-raceme inflorescence, about twice as long as the apical leaf or more, appearing at first sight racemose, with the racemes parallel to the peduncle, each raceme subtended by a bract, petals narrowly spatulate, with a wide reniform, sub-hexagonal bilobed lip, deeply cordate, the sides revolute and thus appearing apron-shaped. It is similar to *Epidendrumgracillimum* Rchb.f. & Warsz. but that species has narrower leaves, fewer flowers, a long narrow cuniculus penetrating about half the ovary, linear petals, and an entire, reniform lip. *Epidendrumphysophorum* Schltr. from Bolivia has a much shorter inflorescence, the branches spreading, the flowers smaller, the inflated cuniculus at the apex of the ovary, the comparatively slenderer column, the petals linear, and the reniform, entire lip with a dentate-erose margin.

### ﻿ Epidendrum echinatiantherum

Taxon classificationPlantaeAsparagalesOrchidaceae

Hágsater, E.Santiago, J.P.Arista & Edquén sp. nov.

75274AB6-95F1-5C07-8A31-14BA19DB86D5

urn:lsid:ipni.org:names:77320357-1

Fig. 6

#### Type material.

**Peru. Amazonas**: Prov. Bongará: Distr. Yambrasbamba: Perla del Imaza, Área de Conservación Privada La Pampa del Burro, Bosque de Piedra, 1682 m, 28 Aug. 2021, *J. P. Arista*, *J. D. Edquén*, *E. Yrigoín & L. Iliquin 151* (holotype: KUELAP!).

#### Diagnosis.

Similar to *Epidendrummadsenii* Hágsater & Dodson, both vegetatively and florally, but the main distinguishing feature is the dark vinaceous anther with the central green vertical rib with a heavily echinate apical, elongate, pyramidal, truncate process, covered by numerous disorganized white bristles (vs. dark brown-black anther without a green vertical rib, and an unornamented apical process, which is laminar, forming an erect, semi-tubular laminar flap, the margins dentate).

#### Description.

Epiphytic, sympodial, branching, pendulous, ***herb***, 8–13 cm long. ***Roots*** ca. 0.7 mm in diameter very thin, basal on primary stems. ***Stems*** 4.5–13 × 2–3 cm, laterally compressed, new stems produced from sub-apical node of previous stem, sometimes from base of primary stem. ***Leaves*** 10–25 per stem, distributed throughout the length of the stems, articulate, twisted at base so as to be on same plane of stem as in *Dichaea*; sheaths 0.4–0.6 × 0.35–0.45 cm, tubular, laterally compressed, ancipitose, minutely rugose, green; leaves 1.1–4.3 × 0.6–1.2 cm, ovate to lanceolate, acute, fleshy, succulent, margins entire, spreading, medium green. ***Spathe*** lacking. ***Inflorescence*** apical, racemose, sub-corymbose, pendulous, from mature stem, sessile, 3-flowered. ***Floral bracts*** 6–8 × 6–7 mm, conduplicate, widely cordiform when spread, dorsally carinate, especially toward apex, embracing. ***Flowers*** 3, more or less simultaneous, pendulous, pale green, sepals tinged pale brown, calli white; fragrance not recorded. ***Sepals*** 9.5 × 3 mm, nearly spreading, free, narrowly lanceolate, acute, 3-veined, lateral sepals dorsally carinate, apex aristate, margins entire, somewhat revolute. ***Petals*** 9 × 1 mm, free, nearly spreading, linear-lanceolate, acute, 1-veined, margins entire, slightly revolute. ***Lip*** 7.5 × 6.0 mm, united to basal half of column, deeply 3-lobed, fleshy, thick, calli 1.1 mm long, basal, digitiform, divergent, disc unornamented, lateral lobes 4.0 × 2.2 mm, transversely ovate, apex narrowly rounded, nearly spreading flat in natural position, at a 45° angle to midline of lip; mid-lobe 4.5 × 2.6 mm, semi-elliptic, apex rounded. ***Column*** 5.5–6.2 mm long dorsally including clinandrium hood, straight, body of column (to rostellum) about half as long as hood. ***Clinandrium hood*** prominent, semi-tubular, somewhat funnel-shaped, margin erose-dentate, anther deep within. ***Anther*** reniform, dark vinaceous with central green vertical rib, with an elongate pyramidal, truncate flap at apex, covered by numerous disorganized white bristles, heavily echinate. ***Pollinia*** 4, lentil-shaped, caudicles soft and granulose. ***Rostellum*** apical, nearly at an 80° angle with axis of column, slit; viscarium semi-liquid. ***Lateral lobes of stigma*** large, transverse, triangular with stigmatic cavity vertical, transverse. ***Cuniculus*** penetrating nearly to base of pedicellate ovary, somewhat wide, forming an elongate ventral vesicle in pedicel. ***Ovary*** 7.5–10 × 2–3 mm including pedicel, terete, straight, inflated nearly to base by elongate vesicle, slightly more inflated basally. ***Capsule*** not seen.

**Figure 6.**
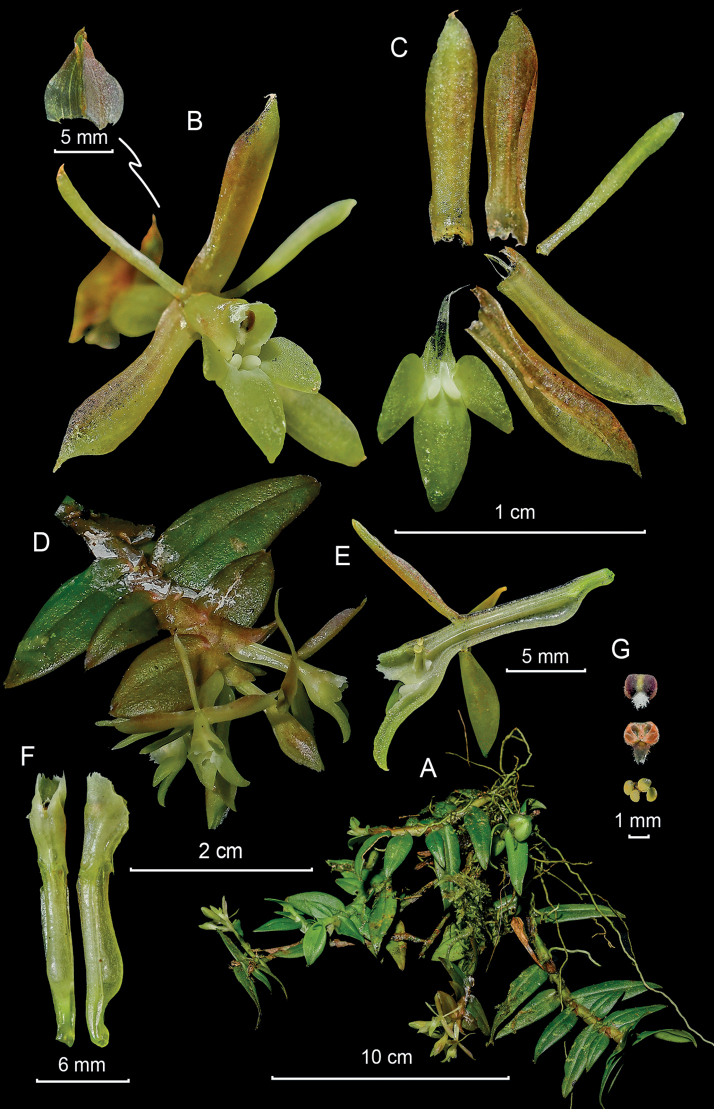
*Epidendrumechinatiantherum* from *Arista et al. 151***A** habit **B** flower and floral bract **C** dissected perianth with dorsal and one lateral sepal shown from above and below, not spread **D** inflorescence at apex of stem **E** longitudinal section of flower **F** column and ovary-pedicel from below (left) and side (right) **G** anther from above (up) and below (down), and pollinarium **H** floral bract (Photographs by J. D. Edquén; plate prepared by A. Cisneros).

#### Distribution.

Known only from the type collection, in Amazonas, Peru, on the eastern slope of the Andes at 1682 m altitude.

#### Habitat and ecology.

Epiphytic in wet montane forest on white sand, covered by accumulated organic material, dominated by palms, with *Cinchona* sp. and *Cedrela* sp. Presence of many Bromeliaceae, mosses. Growing on dry hanging thin branch covered by moss.

#### Phenology.

Flowering in August.

#### Taxonomic notes.

*Epidendrumechinatiantherum* belongs to the Nanum Group, which is characterized by the *Dichaea*-like horizontal or pendulous stems and the inflorescence produced by pairs of opposite flowers without spathes, but with prominent floral bracts. The new species is characterized by the few flowers, the cuniculus forming a long ventral swollen vesicle nearly reaching the base of the pedicellate ovary, relatively large, the sepals 9.5 mm long, the deeply 3-lobed lip with prominent ovate lateral lobes on the lip, the semi-elliptic mid-lobe and the prominent clinandrium hood, somewhat funnel-shaped and especially the reniform anther, dark wine-red with central green vertical rib, with an elongate pyramidal, truncate flap at apex, covered by numerous disorganized white bristles, heavily echinate. It is most similar to *Epidendrummadsenii*, which also has a prominent, very long swollen vesicle at the base of the ovary, nearly as long as the ovary itself, the pale green flowers, the prominent 3-lobed, erose clinandrium hood, the triangular, acute mid-lobe of the lip, and the ornamentation of the anther consisting of a transverse, dentate process in front. It closely resembles *Epidendrumlueri* Dodson & Hágsater, which has pinkish yellow flowers with a bright yellow lip, a denticulate clinandrium-hood, but it is neither 3-lobed nor fimbriate.

#### Etymology.

From Latin *echinatus*, bristly, furnished with numerous rigid hairs, or straight prickles, and *anthera*, the cover of the pollinarium at the apex of the column, which has an appendage heavily covered with white bristles, a rare and prominent feature of this species.

### ﻿ Epidendrum forcipatum

Taxon classificationPlantaeAsparagalesOrchidaceae

C.Schweinf., Fieldiana (Bot.) 33: 36. 1970.

4D116257-0B84-5567-B19F-4D4A6B3B8EAE

Fig. 7

#### Type material.

**Peru.***Sine loc.*, pressed 24 Jul. 1959, *F. Woytkowski s.n.*, cultivated at University of California Botanical Garden at Berkeley 52.1853-1 (holotype: AMES 69508 HUH00070356!; isotypes: AMES 90057 HUH00070357! MO 1785100!); “clonotypes”: pressed 11 Jul. 1962, UC 229015! pressed 15 Mar. 1963, US 2949871 US00093812! Pressed 1 Jul. 1965, UC 1350745!). Pressed by Paul C. Hutchison, 11 Jan. 1961, at University of California Botanical Garden at Berkeley 52.1853, MO 1785407! Pressed 1 Oct. 1963, UC 1350837!

#### Taxonomic synonym.

*Epidendrumpseudoanceps* D.E.Benn. & Christenson, Lindleyana 13(1): 46. (1998). Type: Peru. Huánuco: Leoncio Prado, below El Mirador, 1800 m, 10 Aug. 1966, *D. E. Bennett Jr. 2333* (holotype: AMES!).

#### Description.

Epiphytic, sympodial, caespitose, erect ***herb*** 47–100[215] cm tall. ***Roots*** basal, fleshy. ***Stem*** 25–45[65] × 0.47–0.57 cm, cane-like, ancipitose, erect. ***Leaves*** 6–8, distributed throughout apical half of stem, sub-erect; sheath 1.1–3.0 × 0.47–0.57 cm, tubular, ancipitose, smooth; blade 6–10 × 2.0–2.4[6.4] cm, oblong to elliptic, apex obtuse, coriaceous, dorsally carinate, erect, alternate, margin entire, spreading, somewhat undulate. ***Inflorescence*** 24–65[150] cm long, apical, racemose, flowering repeatedly over several years and producing new racemes from the apical internodes; peduncle 16[34.5] cm long, thin, elongate, ancipitose, totally covered by tubular, acute, bracts, 2–4 cm long; rachis 3.5–4.3[16] cm long, terete, smooth. ***Floral bracts*** 3–5 mm long, much shorter than the ovary, triangular, acuminate. ***Flowers*** 10–17[30] per raceme, simultaneous, resupinate, yellow-ochre, not fragrant. ***Sepals*** 10–12 × 3.3–4.2 mm, free, oblanceolate, slightly concave, subacute, somewhat papillose dorsally toward the apex, 5-veined, fleshy, the ***dorsal*** reflexed, the ***laterals*** spreading to reflexed, somewhat oblique. ***Petals*** [8]10–11 × 0.7–0.8 mm, hanging, free, reflexed, linear, rounded, 1-veined, margin entire, spreading. ***Lip*** [7.5]9.0–9.7 × 10.0–11.4 mm, united to column, 3-lobed, base cordate, margin entire, convex; bicallose, calli laminar, smooth, prolonged into rounded keels, with a central keel running down most of mid-lobe; lateral lobes 4.2–4.7 × 5.5–7.6 mm, dolabriform, with 3 short, thickened keels; mid-lobe 4.2–5.0 × 2.8–3.8 mm, sub-rectangular, emarginate. ***Column*** 6–8.6 mm long, short, thin, straight, dilated toward apex. ***Clinandrium hood*** reduced, entire. ***Anther*** ovoid, with a rounded keel in front, 4-celled. ***Pollinia*** 4, obovoid, laterally flattened; inner pair somewhat smaller; caudicles granulose, longer than pollinia. ***Rostellum*** apical, slit; viscarium semi-liquid. ***Lateral lobes of stigma*** less than ¼ length of stigmatic cavity. ***Cuniculus*** short, slightly penetrating ovary, somewhat inflated behind stigmatic cavity. ***Ovary*** 9.5–10[14] mm long including pedicel, terete, thin, smooth. ***Capsule*** not seen.

**Figure 7.**
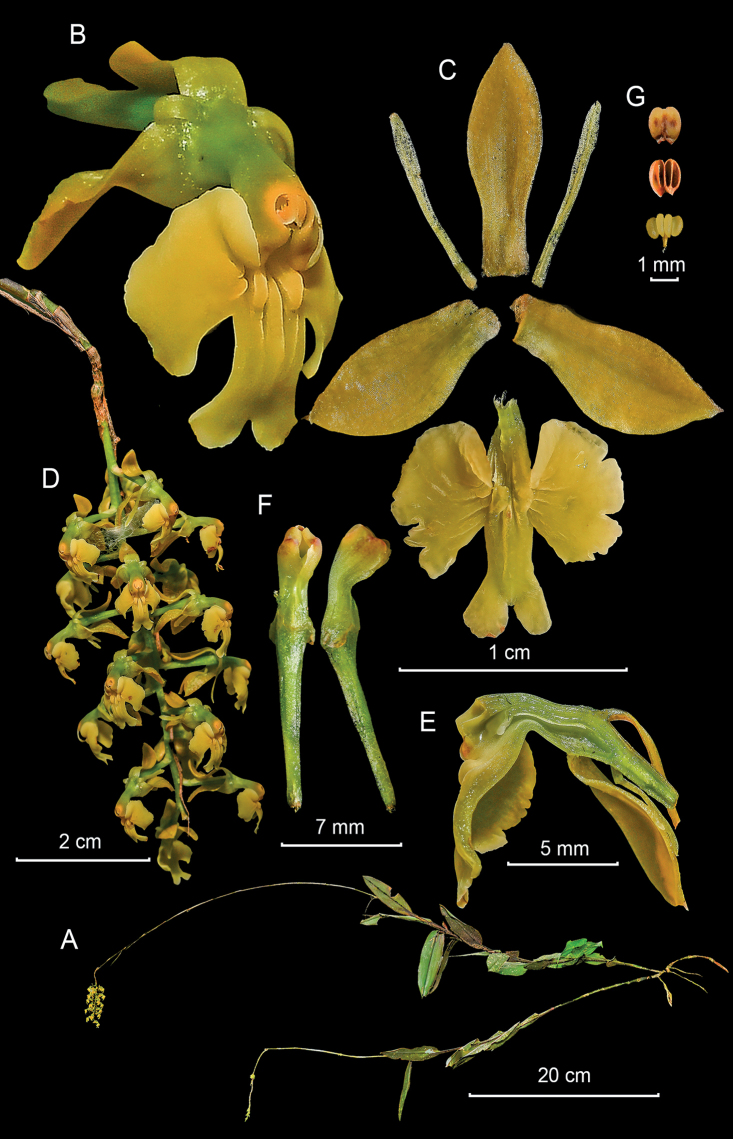
*Epidendrumforcipatum* from *Arista et al. 134***A** habit **B** flower **C** dissected perianth **D** inflorescence **E** longitudinal section of flower **F** column and ovary-pedicel from below (left) and side (right) **G** anther from above (up) and below (down), and pollinarium (Photographs by J. D. Edquén; plate prepared by A. Cisneros).

#### Additional specimens examined.

**Ecuador. Zamora-Chinchipe**: Los Encuentros, Cordillera del “Cóndor”, Mirador, hort, Ecuagenera, 8 Mar. 2003, *H. Medina sub E. Hágsater 13834* (AMO!). **Peru: Amazonas**: Prov. Bongará, Yambrasbamba, Perla de Imaza, Bosque de Piedras, 1682 m, 28 Aug. 2021, *Arista et al. 134* (KUELAP!); **Huánuco**: Leoncio Prado, along road above Cueva de Pavas, 1400 m, 10 Nov. 1991, *Bennett 5354* (MOL! USM, n.v.); **Junín**: Tarma, 6 III 1967, 1500 m, *Bennett 2333* (SEL!) [this specimen has the same collecting number of the type, but a different locality and date; the non-flowering specimen conforms to the species; E. Hágsater, pers. obs.].

#### Other records.

**Peru.***Sine loc.*, received 01 May 2012, *Morón s.n.*, digital image (AMO!); **Junín**: Selva Central, received 03 June 2015, *Torres Paucar s.n.*, digital image (AMO!). **Pasco**: Oxapampa, Huancabamba, P.N. Yanachaga-Chemillén, sector Tunqui-Naciente de la quebrada Muchumayo, 2153 m, 14 Feb. 1009, *R. Vásquez et al.* “*35300 (USM!)*”. **San Martín**: Moyobamba: Quebrada Doncel, 3 Feb. 2021, *Bazan 01*, digital images (AMO!)

#### Distribution.

Known only from the Amazon side of the Andes from southern Ecuador (Cordillera del Cóndor, Zamora-Chinchipe) and northern and central Peru, at 1500–1800 m.

#### Habitat and ecology.

Epiphytic, growing on tree trunk in 20–30 m tall lower montane forest, primary sclerophyllous forest, and montane humid forest with *Cinchona* sp. and *Cedrela* sp.

#### Phenology.

Flowering from March to November

#### Taxonomic notes.

The square brackets in the description indicate the unusually large specimens prepared from cultivated material in the greenhouses of the University of California Botanical Garden at Berkeley, and not seen in wild-collected material. Therefore, they are indicated separately. The original description is based on these measurements.

*Epidendrumforcipatum* belongs to the Anceps Group and Polyanthum Subgroup, recognized by the caespitose habit, the simple stems, the pluriracemose inflorescence, flowering over several years, the elongate racemes and the fleshy flowers and linear petals. The species is recognized by the generally crisped-undulate leaves, about 10–17[30] flowers, green-ochre in color, and filiform petals. *Epidendrumforcipatoides* Hágsater from Bahia and Minas Gerais, Brazil attains a smaller size and bears leaves with non-undulate margins and smaller flowers, the obovate sepals being about 6.0–6.5 mm long.

### ﻿ Epidendrum imazaense

Taxon classificationPlantaeAsparagalesOrchidaceae

Hágsater, E.Santiago, J.P.Arista & Edquén sp. nov.

73613CA1-5AA2-5537-BD1C-252BA5F63DD6

urn:lsid:ipni.org:names:77320358-1

Fig. 8

#### Type material.

**Peru. Amazonas**: Prov. Bongara, Distr. Yambrasbamba, camino a la Perla del Imaza, 1886 m, 17 Jul. 2022, *J. P. Arista*, *J. D. Edquén*, *E. Hágsater*, *E. Santiago*, *G. A. Salazar*, *E. Yrigoín*, *L. I. Cabrera & K. Edquen 272* (holotype: KUELAP!).

#### Diagnosis.

Similar to *Epidendrumfreireanum* Hágsater & E.Santiago, but the plants are smaller, 3.6–10 cm tall (vs. plants 10–40 cm tall), the leaves 1.1–3.6 cm long (vs. leaves 3.7–7.0 cm long), the ovary 10 mm long, sigmoid, with a prominent ventral vesicle, inflated in the middle (vs. ovary 6 mm, straight, not inflated), the sepals 5.5 mm long (vs. sepals 10 mm long), and the flowers pale green with a red-brown tinge on the tepals (vs. flowers purple brown with lip and apex of column ivory white).

**Figure 8.**
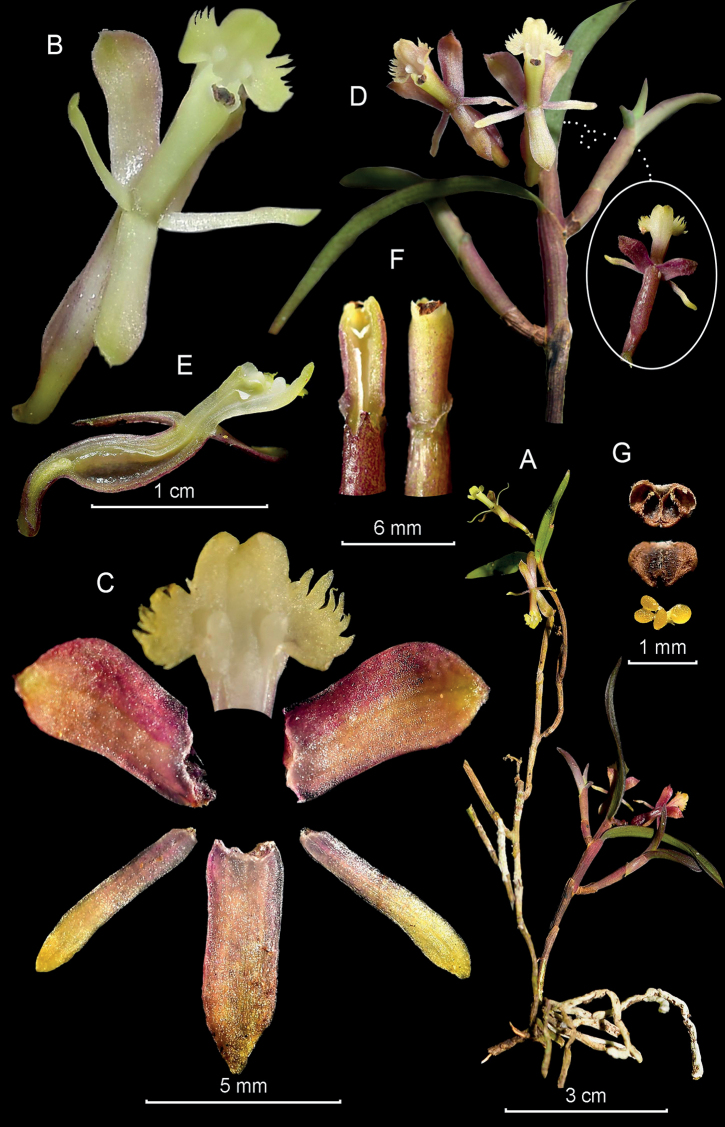
*Epidendrumimazaense* from *Arista et al. 272***A** habit **B** flower **C** dissected perianth **D** inflorescence at apex of stem **E** longitudinal section of flower **F** column and ovary apex from below (left) and above (right) **G** anther from below (up) and above (down), and pollinarium (Photographs by J. D. Edquén; plate prepared by X. Alcántara).

#### Description.

Epiphytic, monopodial, branching, erect ***herb***, ca. 10 cm tall, with new stems produced from upper internodes of previous stem. ***Roots*** ca. 2 mm in diameter, from base of primary stem only, fleshy, thick. ***Stems*** 2–4 × 0.1–0.25 cm, simple, cane-like, produced from sub-apical internodes of previous stem, sometimes two from same stem, thin, laterally compressed, erect, straight or slightly arched upward, base covered by sheaths 1–10 mm long, tubular, non-foliar. ***Leaves*** 2–3, aggregate toward apex of stem, alternate, articulate, coriaceous, slightly conduplicate; sheaths 7–8 × 1.2–2.5 mm, tubular, minutely striated, red; blade 1.1–3.6 × 0.25–0.5 cm, lanceolate, acute, minutely apiculate, margin entire, spreading. ***Spathe*** lacking. ***Inflorescence*** ca. 2 cm long, apical, racemose, few-flowered; peduncle 1 cm long, sub-terete, rachis 0.3 cm long. ***Floral bracts*** 1.5–2.8 mm long, much shorter than ovary, triangular, long acuminate, embracing. ***Flowers*** 3, simultaneous, non–resupinate, tepals pale green turning yellow tinged red brown ventrally, dorsally red brown, lip and column pale green to yellow, concolor; fragrance not registered. ***Ovary*** 10 mm long, sigmoid, with a prominent ventral vesicle inflated toward middle and involving 2/3 of ovary length, verrucose. ***Sepals*** free, 3-veined, dorsally verrucose, margins entire, spreading; dorsal sepal 5.5 × 2.0 mm, reflexed, oblong, obtuse; lateral sepals 5.5. × 2.5 mm, partly spreading, narrowly obovate, oblique, obtuse, minutely apiculate. ***Petals*** 5 × 1 mm free, spreading, oblong, obtuse, 1-veined, margins entire, spreading. ***Lip*** 3.0–3.3 × 5.5 mm, totally united to column, 3-lobed, cordate at base; bicallose, calli spherical, widely spaced, disc with a single mid-rib, thickened and reaching apical sinus; lateral lobes 1.3–1.7 × 2.2–2.4 mm, dolabriform with apical margin fimbriate; mid-lobe 1.5 × 2.3 mm, rectangular apex slightly emarginate. ***Column*** 4.5–5.0 mm long, thin, straight. ***Clinandrium hood*** short, margin entire. ***Cuniculus*** penetrating 2/3 of ovary, inflated in middle of ovary, forming an elongate prominent vesicle, unornamented. ***Anther*** reniform, 4-celled. ***Pollinia*** 4, obovoid, laterally compressed. ***Rostellum*** apical, slit; viscarium semi-liquid. ***Lateral lobes of stigma*** prominent, half as along as stigmatic cavity. ***Ovary*** 10 mm long including pedicel, sigmoid, with a prominent ventral vesicle inflated toward middle and involving 2/3 of ovary length, verrucose, furrowed. ***Capsule*** not seen.

#### Distribution.

Presently known only from the type from northern Peru, department of Amazonas, eastern range of the Andes around the ACPPB, at 1886 m.

#### Habitat and ecology.

Epiphytic in relict wet montane forest.

#### Phenology.

Flowering in July.

#### Taxonomic notes.

*Epidendrumimazaense* belongs to the Fruticetorum Group, which is characterized by the monopodial, branching habit, the few-leaved stems, flattened above, the erect to arching racemose to pluri-racemose inflorescence producing new racemes from the same peduncle in successive years, the flowers generally non-resupinate, and the 3-lobed lip with the distal margins of the lateral lobes dentate to shortly fimbriate. The new species is recognized by the short inflorescences, nearly sessile, with 3 non-resupinate flowers, yellow tepals, ventrally tinged red-brown, dorsally red-brown; lip and column pale green, concolor, the sepals 5.5 mm long, dorsally verrucose and the ovary sigmoid with a prominent, elongate vesicle in the middle of the ovary. *Epidendrumfreireanum* has purple leaves and stems, smaller flowers, the sepals being 10 mm long, the sepals, petals and basal half of column vinaceous to purple-brown, apical half of column and the lip ivory white, immaculate, and the lip with a single wide mid-rib. *Epidendrumfruticetorum* Schltr. (see Cisneros and Hágsater in Hágsater and Santiago 2019: t. 1721) is similar but larger in habit and flowers, the sepals are 14 mm long, the flowers are green, the lip with 3 narrow parallel ribs is pale green and the column green. Bennett and Christenson (1995a: t. 247) misidentified their plate of *Epidendrumfruticetorum* as *E.odontospathum* Schltr.

#### Etymology.

In reference to the Imaza river, whose basin includes the Pampa del Burro. This river is a tributary of the Chiriacu river, an affluent of the Marañón river.

### ﻿ Epidendrum mavrodactylon

Taxon classificationPlantaeAsparagalesOrchidaceae

Hágsater, Edquén & E.Santiago, Icon. Orchid. 16(2): t. 1682. 2018.

201CA038-E6A5-5F6E-984D-9DD6BC168CB9

Fig. 9

#### Type material.

**Peru. San Martín**: Prov. Rioja, correspondiente al área natural protegida Bosque de Protección Alto Mayo-BPAM, Sector Venceremos, Zona 18, 1807 m, 30 Jan. 2018, *J. D. Edquén 401* (holotype: HURP!).

#### Description.

Epiphytic, caespitose, sympodial, compact, small, reclining ***herb***, 3 cm tall. ***Roots*** 1 mm in diameter, basal along rhizome, thin, white. ***Stems*** 1.3–2 × 0.3–0.4 cm, cane-like, simple, laterally compressed, somewhat reclining. ***Leaves*** 4–7, fleshy, distichous, erect, semi-terete, concave, arching and partially imbricated, somewhat conduplicate, dark green, concolor; sheath 1–3 mm long, tubular, narrow at base gradually widened toward apex; blade 0.5–1.4 × 0.2–0.4 cm, narrowly lanceolate obtuse to sub-acute, minutely apiculate, margin entire, dentate at apex. ***Spathe*** 1, 5–9 × 5–7 mm when spread, elliptic, obtuse, minutely apiculate, conduplicate. ***Inflorescence*** apical, sessile, single-flowered. ***Floral bract*** not seen (hidden within spathe). ***Flower*** 1, resupinate yellowish green, sepals and petals with a bronze tinge; fragrance not registered. ***Sepals*** 8–10 × 3.0–4.0 mm, apex acuminate, minutely apiculate, 3-veined; dorsal sepal free, partly spreading, nearly parallel to column, narrowly lanceolate, margin minutely papillose toward apex, spreading; lateral sepals obliquely untied to base of column, spreading, narrowly ovate-triangular, oblique, margin entire, revolute. ***Petals*** 8–8.5 × 0.5 mm, free, partly spreading, parallel to dorsal sepal, linear, falcate, acute, 1-veined, margin entire, spreading. ***Lip*** 7.0–7.2 × 4–5 mm, united to column, entire, elliptic, base cuneate, apex acute, minutely apiculate, margin slightly erose; bicallose, calli globose, prominent; disc with a very low midrib. ***Column*** 4 mm long, thin at base, gradually wider toward apex, triangular in lateral view. ***Rostellum*** apical, slit; viscarium semi-liquid, white. ***Clinandrium hood*** prominent, slightly longer than body of column, totally covering anther, margin erose. ***Anther*** reniform, 4-celled. ***Pollinia*** 4, obovate, complanate, convex-flat, caudicles as long as pollinia. ***Lateral lobes of stigma*** not seen. ***Cuniculus*** inflated ventrally along apical half of ovary. ***Ovary*** 11–15 mm long, ventrally inflated, forming a prominent, elongate vesicle along apical, ventral half of ovary. ***Capsule*** not seen.

**Figure 9.**
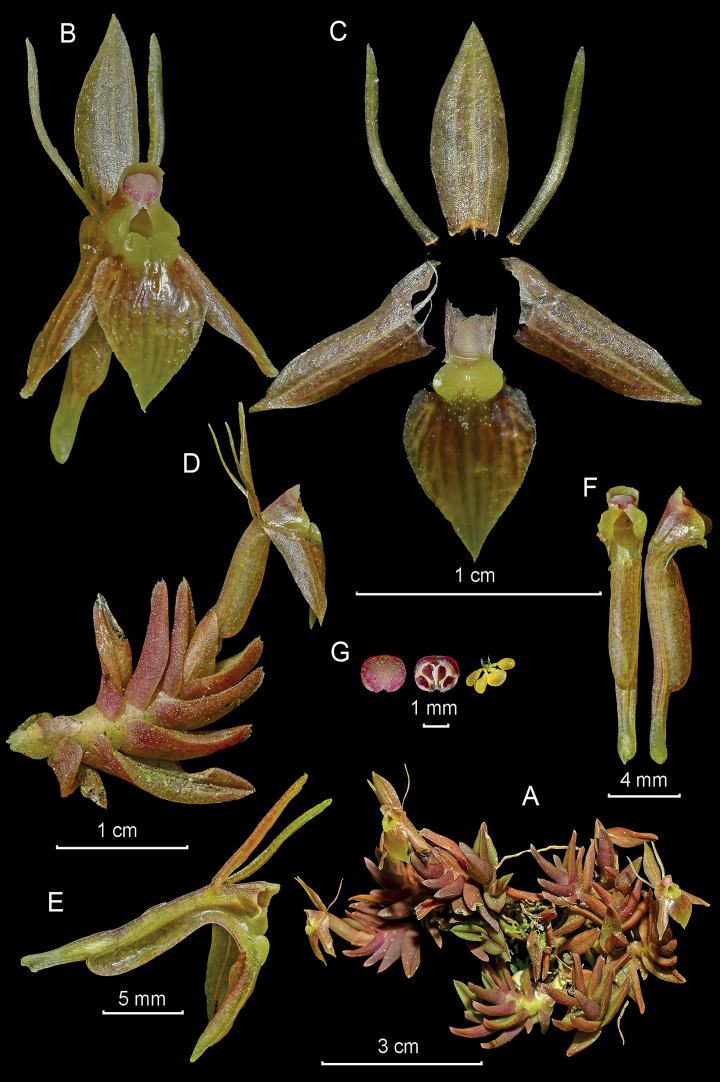
*Epidendrummavrodactylon* from *Arista et al. 156***A** habit **B** flower **C** dissected perianth **D** flower at apex of stem **E** longitudinal section of flower **F** column and ovary-pedicel from below (left) and side (right) **G** anther from above (left) and below (right), and pollinarium (Photographs by J. D. Edquén; plate prepared by A. Cisneros).

#### Additional specimens examined.

**Peru. Amazonas**: Prov. Bongará, Distr. Yambrasbamba, Perla del Imaza, 1763 m, 28 Aug. 2021, *Arista & Edquén 156* (KUELAP!).

#### Other records.

**Peru.** Cultivated in Moyobamba in the private collection of José Altamirano, 16 May 2009, digital images, *Jenny s.n.* (AMO!).

#### Distribution.

Known presently only from northern Peru, from the limits between the departments of San Martín and Amazonas. On both sides of the ridge that forms the border of the two departments. In montane wet forest at 1763–1800 m elevation.

#### Habitat and ecology.

Epiphytic on *Erythrina* L. Ground with abundant moss, and accumulation of organic matter.

#### Phenology.

Flowering in January to August.

#### Taxonomic notes.

*Epidendrummavrodactylon* represents an addition to the orchids of the department of Amazonas. It belongs to the Porpax Group, which is characterized by the sympodial, somewhat repent habit, usually forming large mats; the short, cane-like stems with very fleshy, succulent, articulate, distichous leaves, the apical margin minutely ciliate; the single-flowered inflorescence with sessile flowers; and the lip generally reddish purple. *Epidendrummavrodactylon* is recognized by the very small plants, up to 3 cm tall, with leaves 0.5–1.4 cm long, semi-terete, concave and arching, flowers yellowish green, sepals and petals tinged bronze; dorsal sepal and petals nearly parallel to the column, lip 7.0–7.2 × 4–5 mm, elliptic, acute, and the ovary with a prominent ventral, elongate vesicle, half as long as the ovary. *Epidendrumalthaniorum* Hágsater & Collantes from Cusco has larger plants, 4.5–10 cm tall, leaves 1.3–5 cm long, straight, flowers lime-yellow, lip orbicular, rounded with a heart-shaped red blotch in the middle, and the ovary not inflated, not forming a vesicle. *Epidendrumneolehmannia* Schltr. of the same group has an equally inflated vesicle ventrally along the ovary caused by the inflated cuniculus, but the flowers have a cordiform lip 12–15 × 12–17 mm, with two parallel calli at the base, no obvious mid-rib, and the leaves are semi-terete, 1.0–2.5 × 0.4–0.7 cm.

### ﻿ Epidendrum ochrostachyum

Taxon classificationPlantaeAsparagalesOrchidaceae

Hágsater, E.Santiago, J.P.Arista & Edquén sp. nov.

E87A2BB0-74A4-5E78-A12E-4C38639C63AF

urn:lsid:ipni.org:names:77320359-1

Fig. 10

#### Type material.

**Peru. Amazonas**: Prov. Bongará, Distr. Yambrasbamba, Perla del Imaza, Río Rojo, Pampa del Burro, 1839 m, 25 Aug. 2021, *J. P. Arista*, *J. D. Edquén*, *E. Yrigoín & L. Iliquín 79* (holotype: KUELAP!).

#### Diagnosis.

Similar to *Epidendrumbangii* Rolfe but differs by the shorter lateral sepals, 13 mm long, spreading, ovate-elliptic, the apex obtuse (vs. lateral sepals 16–18 mm long, slightly reflexed, obliquely ovate, with the apex acuminate), the shorter petals, 10 mm long, oblanceolate, apex obtuse (vs. petals 13–15 mm long, linear, apex acuminate), the floral bracts 8–13 mm long, nearly as long as the ovary (vs. floral bracts 11–22 mm long, longer than the ovary), and the column with a pair of truncate wings (vs. column with a pair of rounded wings).

#### Description.

Terrestrial, monopodial, branching, erect ***herb***, ca. 66 cm tall including inflorescence. ***Roots*** 1.0–2.5 mm in diameter, basal, scarce, fleshy. ***Stems*** cane-like, erect, straight, scarcely branching sub-apically, primary stem 54 × 0.6 cm, branches 7 × 0.3 cm; base covered by sheaths 6–30 mm long, tubular, non-foliar, scarious. ***Leaves*** 9 on primary stem, 3–5 on branches, distributed throughout the stems, articulate, slightly conduplicate, spreading, coriaceous, rigid, medium green on both sides, margins red-brown; sheaths 0.5–5.0 × 0.3–0.6 cm, tubular, minutely striated, rugose, red; blade 2.5–12 × 0.6–2 cm, lanceolate, acute, margin entire, spreading. ***Spathe*** lacking. ***Inflorescence*** 12 cm long on primary stem, ca. 6 cm long from branches, apical, racemose, erect, laxly few-flowered; peduncle 1.2 cm long, terete, without bracts, red-brown; rachis 5–11 cm long. ***Floral bracts*** 8–13 × 5–7 mm, nearly as long as ovary, widely ovate, sub-acuminate, oblique, embracing. ***Flowers*** up to 15, successive, non-resupinate, fleshy, ochre-yellow to olive green, sometimes tinged orange, dorsal surface of sepals red-brown to wine-brown, lip yellow; fragrance not registered. ***Sepals*** free, spreading, ovate to ovate-elliptic, obtuse, minutely apiculate, 3-veined, margins entire, spreading; dorsal sepals 12 × 5 mm, lateral sepals 13 × 7 mm. ***Petals*** 10 × 3 mm, free, spreading, oblanceolate, obtuse, 1-veined, margin entire, spreading. ***Lip*** 8 × 10 mm, united to column, entire, widely cordiform, base deeply cordate, apex acute, embracing apex of column in natural position; ecallose, strongly pubescent in front of stigmatic cavity. ***Column*** 8 mm long, thick, apex bidentate, with a pair of truncate wings. ***Clinandrium hood*** reduced, margin entire. ***Anther*** ovoid, apex acute, 4-celled. ***Pollinia*** 4, obovoid, laterally compressed; caudicles soft and granulose. ***Rostellum*** apical, slit; viscarium semi-liquid. ***Lateral lobes of stigma*** small, ¼ length of stigmatic cavity. ***Cuniculus*** shallow, without penetrating pedicellate ovary, strongly pubescent in front of stigmatic cavity. ***Ovary*** 8–9 mm long, terete, thick, furrowed. ***Capsule*** not seen.

**Figure 10.**
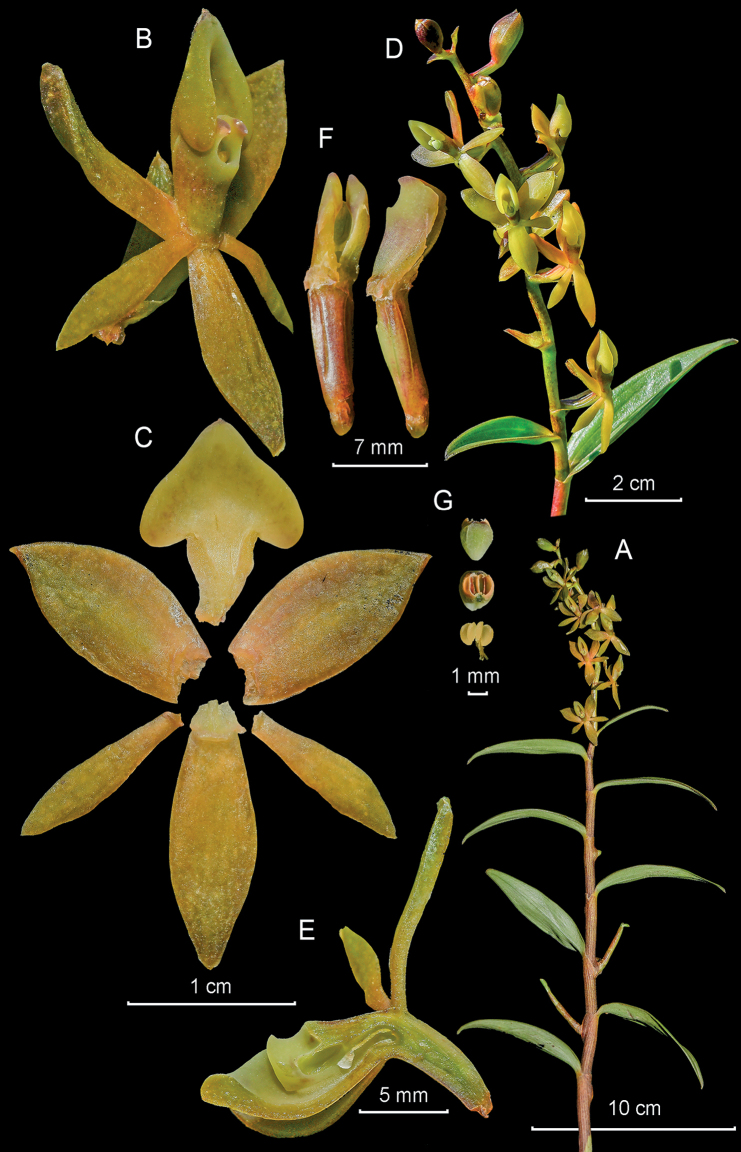
*Epidendrumochrostachyum* from *Arista et al. 79***A** habit **B** flower **C** dissected perianth **D** inflorescence at apex of stem **E** longitudinal section of flower **F** column and ovary-pedicel from below (left) and side (right) **G** anther from above (up) and below (down), and pollinarium (Photographs by J. D. Edquén; plate prepared by A. Cisneros).

#### Additional specimens examined.

**Peru. Amazonas**: Prov. Bongará, Distr. Yambrasbamba, La Perla Vieja, Pampa del Burro, 1880 m, 22 Aug. 2021, *Arista et al. 12* (KUELAP!); *ibid. loc.*, 1850 m, 22 Aug. 2021, *Arista et al. 61* (KUELAP!); Jumbilla–Molinopampa, 2500–2900 m, 6 Nov. 2012, *van der Werff 25206* (HOXA! USM!).

#### Other records.

**Peru. Amazonas**: Prov. Bongará, Distr. Florida, Pomachapanda, 2300–2500 m, 8 Nov. 2010, *Dalström 3178*, digital image (AMO!); Bosque Protector Alto Nieva, 2184 m, 17 Mar. 2019, *Hágsater 16077*, digital image (AMO!); **San Martín**: Prov. Rioja, Distr. Pardo Miguel Naranjos, Sector Venceremos, 1959 m, 23 Feb. 2017, *Edquén 2019*, digital images (AMO!); *ibid. loc.*, 1715 m, 21 Feb. 2017, *Edquén 2020*, digital images (AMO!); *ibid. loc.*, 1730 m, 22 Feb. 2017, *Edquén 2021*, digital images (AMO!); *ibid. loc.*, 1975 m, 20 Feb. 2017, *Edquén 2022*, digital images (AMO!); *ibid. loc.*, 1918 m, 6 Nov. 2015, *Edquén 2023*, digital images (AMO!).

#### Distribution.

Known only from northern Peru, on the border between the departments of Amazonas and San Martín, growing at 1715–2500 m.

#### Habitat end ecology.

Terrestrial, on road bank, in white sand with abundant organic matter 10 cm deep, in low forest.

#### Phenology.

Flowering in August to March.

#### Taxonomic notes.

*Epidendrumochrostachyum* belongs to the Macrostachyum Group, which is characterized by the monopodial plants with sub-apical branching, the rugose leaf sheaths, the large leaves, generally spreading, the erect racemose inflorescence, the fleshy flowers generally green to black (ripe olive colored), as well as yellow to pink to purple, with an entire, conduplicate, generally ecallose lip embracing the column, and the cuniculus pubescent to papillose in front of the stigmatic cavity and at least at the base of the lip. The species is recognized by the small plant (up to 66 cm tall), leaves 2.5–12 × 0.6–2 cm, lanceolate, acute, the inflorescence of primary stem with up to 15 flowers, the floral bracts nearly as long as the ovary, the flowers ochre-yellow to olive green, sometimes tinged orange, dorsal surface of sepals red-brown to wine-brown, lip yellow, the lateral sepals 13 mm long, spreading, petals oblanceolate, obtuse, the lip deeply cordiform, acute, and the column with a pair of truncate wings. *Epidendrumbangii* is vegetatively similar, but the floral bracts are longer than the ovary, the flowers are purple, the lateral sepals 16–18 mm long, the petals are linear, acuminate, the lip is cordiform, and the column has a pair of rounded apical wings. *Epidendrumodontostachyum* Hágsater & E.Santiago has larger plants, ca 1 m tall, leaves 6–8 × 0.6–1.6 cm, linear-lanceolate, the inflorescence up to 20 cm long, the flowers dark green with the lip purple-green, the petals 12 × 1.5 mm, slightly reflexed, and the floral bracts triangular and acuminate.

#### Etymology.

From the ochre color of the flowers, from the Greek ὠχρός, and σταχυς, from the Greek spike, in reference to the Macrostachyum Group to which the species belongs.

### ﻿ Epidendrum olorteguii

Taxon classificationPlantaeAsparagalesOrchidaceae

Damián, Hágsater & Mitidieri, Phytotaxa 552(1): 100. 2022.

361CF378-8221-54BE-AD0C-744BF654ED5C

Fig. 11

#### Type material.

**Peru. Amazonas**: Prov. Bongará: Distr. Yambrasbamba: Centro Poblado El Progreso, 2200–2300 m, Nov. 2020, *S. Olortegui & A. Damián 5050* (holotype: USM!; isotype: UFV!).

#### Description.

Epiphytic, sympodial, scandent, erect ***herb***, up to 120–140 cm tall, new stems produced from middle of previous stem. ***Roots*** 5–6 mm in diameter, thick, scarce, from base of primary stems. ***Stems*** 14–45 × 0.9–1.3 cm, simple, cane-like, terete, new stem produced from sub-apical internode of previous stem, lower part covered by 3–5 tubular, imbricate, chartaceous, gray non-foliar sheaths. ***Leaves*** 2–5 aggregate toward apex apical half of stem, distichous, articulate, spreading, base embracing; sheaths 1.4–3.5 × 0.9–1.3 cm, tubular, infundibuliform in dry specimens; blade 6.0–14.5 × 2.2–6.0 cm, elliptic to elliptic-lanceolate in mature specimens, acute, thin, margin entire. ***Spathe*** lacking. ***Inflorescence*** 16–20 cm long, apical, racemose, arching nutant, few-flowered; peduncle 3–5 cm long, laterally compressed, slightly ancipitose, rachis 12–15 cm long, sub-terete. ***Floral bracts*** 1.0–2.3 × 0.6–0.8 cm, slightly shorter than ovary, progressively shorter toward apex of rachis, triangular, acuminate, margins microscopically denticulate. ***Flowers*** 4–13, flowers successive, 2–5 open at a time, resupinate, green to yellow or rarely ivory white (*Léon Martínez s.n.*), column darker green or dirty white, tinged purple toward apex, lip when green pale toward disc and calli; fragrance not detected. ***Sepals*** spreading, fleshy, slightly convex, free, 5-veined, margins entire, spreading; dorsal sepal 30–38 × 9–12 mm, lanceolate to oblong-lanceolate, acute, basal margins revolute, slightly carinate dorsally; lateral sepals 32–40 × 8–13 mm, lanceolate to oblong-lanceolate, oblique, acuminate, with a prominent dorsal keel. ***Petals*** 27–35 × 5–7 mm, strongly reflexed, parallel to ovary, fleshy, narrowly oblanceolate, acuminate, 3-veined, margins entire, spreading. ***Lip*** 22–32 × 28–30 mm, fleshy, trilobed, fused to column, base obliquely cordate; bicallose, calli divergent, elongate, rounded, disc 3-ribbed, lateral ribs in front of calli inconspicuous, low, parallel, with a low, wide mid-rib reaching apex of labellum; lateral lobes 10–15 × 10–14 mm, prominent, convex, transversely sub-rectangular, basal corners narrowly rounded to obtuse, distal corner widely rounded, multi-veined, margins entire, slightly revolute; mid-lobe 8–13 × 20–22 mm, curved in natural position, flabellate with two obliquely triangular lobes and a narrow, cuneate isthmus in basal half, apical half truncate, with a short, thickened, narrowly triangular, reflexed, apiculus at apex, lobes divergent, obliquely triangular, obtuse, slightly revolute at apex, margins entire. ***Column*** 14–16 mm long, short, thick, straight, slightly widening apically, constricted near base. ***Clinandrium hood*** very short, margin entire. ***Anther*** ovoid, glandular-papillose, 4-celled. ***Pollinia*** 4, dark yellow, obovate, laterally compressed, subequal, caudicles granulose, as long as pollinia. ***Rostellum*** apical, slit; viscarium semi-liquid, translucent. ***Lateral lobes of stigma*** small, 1/5 length of stigmatic cavity. ***Cuniculus*** deep, penetrating two thirds of ovary, widened behind perianth, unornamented. ***Ovary*** 16–28 mm long, slightly arching, terete, furrowed, ventrally thickened apical third. ***Capsule*** ellipsoid, 6.3 cm long, pedicel 3 mm long, body 4 × 3 cm, apical neck 2 cm long.

**Figure 11.**
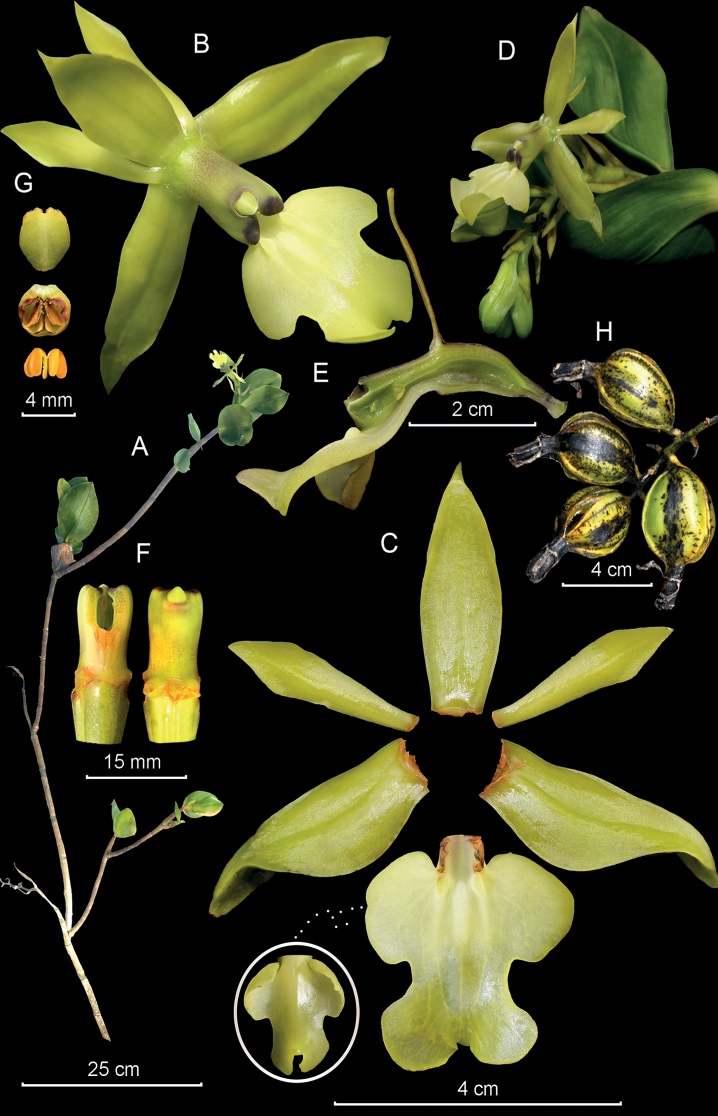
*Epidendrumolorteguii* from *Arista et al. 271***A** habit **B** flower **C** dissected perianth **D** inflorescence at apex of stem **E** longitudinal section of flower **F** column and ovary apex from below (left) and above (right) **G** anther from above (up) and below (down), and pollinarium **H** capsules (Photographs by J. D. Edquén; plate prepared by X. Alcántara).

#### Additional specimens examined.

**Peru. Amazonas**; Prov. Bongará, Distr. Yambrasbamba, centro poblado El Progreso, 2300 m, Nov. 2017, *Olórtegui s.n.* (USM!); Distr. Buenos Aires, centro poblado Santa Rosa, 2079 m, 15 Jul. 2022, *Arista et al. 271* (KUELAP!).

#### Other records.

**Peru. Amazonas**: Prov. Bongará: Distr. Yambrasbamba: El Progreso, Vivero “Mi Angelito,” colectada en el área de Nicolasa Velásquez, white form, *Martínez s.n.*, digital image (AMO!); km 5.5, camino a la Perla del Imaza, 2083 m, 15 Jul. 2022, *Hágsater 16418*, digital images (AMO!); La Esperanza, hort. vivero alto, Orquídeas Amazónicas, 16 Jul. 2022, *Hágsater 16490*, digital images (AMO!); **San Martín**: Prov. Rioja, Distr. Pardo Miguel Naranjos, Chisquilla, 2100 m, 22 June 2019, *Edquén 2092*, digital images (AMO!).

#### Distribution.

*Epidendrumolorteguii* is presently known from two localities 50 km distant from each other, on the eastern slopes of the Andes in northern Peru, at 2100–2300 m.

#### Habitat and ecology.

Epiphytic in humid montane forests.

#### Phenology.

Flowering from July through November.

#### Taxonomic notes.

*Epidendrumolorteguii* belongs to the Incomptum Group which is characterized by having erect successive lateral growths produced from the middle of the previous growth, few leaves aggregated toward the apex of the stems, the short apical inflorescence with fleshy green to violet-green flowers with short ovaries, and the lip entire to 3-lobed. *Epidendrumolorteguii* is recognized by its large habit, the large leaves, 6.0–14.5 × 2.2–6.0 cm, the large green flowers to yellow or rarely ivory white, column darker green or dirty white, tinged purple toward apex, the lip cream suffused with light green at the mid-lobe, the lanceolate to oblong-lanceolate sepals 30–40 mm long, the petals narrowly elliptic, and the lip 22–32 × 28–30 mm, 3-lobed, the lateral lobes 10–15 × 10–14 mm, prominent, transversely sub-rectangular, basal corners narrowly rounded, distal corner widely rounded and the mid-lobe 8–13 × 20–22 mm, constricted at base, then transversely elliptic, sides involute; apex with a short, thickened, narrowly triangular, reflexed apiculus. It is somewhat similar to *Epidendrumtamaense* Foldats found from Ecuador to Venezuela, which has smaller plants, leaves 3–14 × 2.0–4.3 cm, green to olive-green flowers, lip marked with purple veins; sepals 15–21 mm long; petals narrowly obtrullate, obtuse to acute, and the 3-lobed lip has lateral lobes sub-orbicular, mid-lobe obcuneate, deeply emarginate, with two sub-orbicular lobes.

### ﻿ Epidendrum parvireflexilobum

Taxon classificationPlantaeAsparagalesOrchidaceae

Hágsater, J.P.Arista & Edquén sp. nov.

0B1912EF-BBAE-50A1-8A5E-28D041E4FEA9

urn:lsid:ipni.org:names:77320360-1

Fig. 12

#### Type material.

**Peru. Amazonas**: Prov. Bongará: Yambrasbamba: Perla del Imaza, La Perla Vieja, Pampa del Burro, 1871 m, 22 Aug. 2021, *J. P. Arista*, *J. D. Edquén*, *E. Yrigoín*, *L. Iliquin 49* (holotype: KUELAP! [LCDP voucher]).

#### Diagnosis.

Similar to *Epidendrumreflexilobum* C.Schweinf., but overall smaller, with plants 22–46 cm tall including the inflorescence (vs. 40–120 cm tall), sepals 9.5–10.8 mm long, elliptic (vs. sepals 12.5–13.5 mm long, obovate-elliptic), the lateral lobes of the lip semi-ovate, erect (vs. lateral lobes spreading, twisted 90° in natural position, narrowly obovate), the callus 3-ribbed, forming a cuneate platform only reaching middle of mid-lobe (vs. callus 3-ribbed, ribs not forming a platform, with the mid-rib longer and reaching apical sinus).

#### Description.

Epiphytic, sympodial, caespitose, erect ***herb***, 22–46 cm tall including inflorescence. ***Roots*** 2 mm in diameter, basal, terete, fleshy, white. ***Stems*** 8–22 × 0.3 cm, simple, cane-like, terete to slightly compressed toward apex, thin, basal half covered by non-foliar sheaths. ***Leaves*** 7–12, distichous, distributed along upper 1/4 of stem; sheaths 4.4–6.0 × 0.4 cm, tubular, smooth, vinaceous, papyraceous when dry; blade 4.0–7.8 × 0.8–2.8 cm, oblong, apex unequally bilobed, articulate, coriaceous, smooth, medium green on both sides, margins entire. ***Spathe*** lacking. ***Inflorescence*** 12–33 cm long, racemose to pluri-racemose, laxly flowered, cylindric; peduncle 10–28 cm long, elongate, covered by numerous tubular, imbricate bracts 4.5 × 0.4 cm, acute, scarious when dry, striated, papyraceous; rachis ca. 4.8 cm long. ***Floral bracts*** 1–3 × 0.7–3.0 mm, much shorter than ovary, decreasing in size toward apex, triangular, acuminate to acute, embracing. ***Flowers*** ca. 6–12, per raceme, successive, 3–6 open at a time, non-resupinate, pale to bright red, callus yellow; fragrance none. ***Sepals*** spreading, apex obliquely rounded, short apiculate, 7-veined, margin entire, spreading; dorsal sepal 9.5–10.2 × 4.5–4.9 mm, elliptic; lateral sepals 9.9–10.8 × 5.0–5.5 mm, elliptic, oblique, sub-obtuse. ***Petals*** 10.6–13.5 × 4.0–5.0 mm, extended, oblanceolate or cuneate-spathulate, apex acute, 3–5-veined, margin entire, spreading. ***Lip*** 5.7–7.8 × 8.2–9.0 mm, united to column, deeply 3-lobed, in natural position mid-lobe flat, extended, lateral lobes sub-erect, base cordate, distal margins irregularly laciniate; callus low, rectangular, truncate to rounded, with mid-rib formed by 3 straight, parallel ribs on mid-lobe, mid-rib longer, with two divergent bifid calli at base of lateral lobes; lateral lobes 3.0–3.5 × 4.2–5.9 mm, semi-obovate, erect in natural position; mid-lobe 4.6 × 8.2 mm, obcuneate, bifid, slightly divergent, deeply and narrowly emarginate. ***Column*** 4.0 mm long, straight, wider at apex, with a pair of long, apical recurved fleshy wings with distal margin erose. ***Clinandrium hood*** very short, margin entire, leaving anther totally exposed. ***Anther*** ovoid, apiculate, surface rugose, 4-celled. ***Pollinia*** 4, narrowly obovoid, laterally compressed, caudicles formed by a pile of elongate pollen tetrads like a pile of tiles. ***Rostellum*** apical, split; viscarium semi-liquid. ***Lateral lobes of stigma*** short, occupying ¼ length of stigmatic cavity. ***Cuniculus*** penetrating half pedicellate ovary, minutely papillose. ***Ovary*** 20 mm long, terete, thin, not inflated, green to red, furrowed. ***Capsule*** not seen.

**Figure 12.**
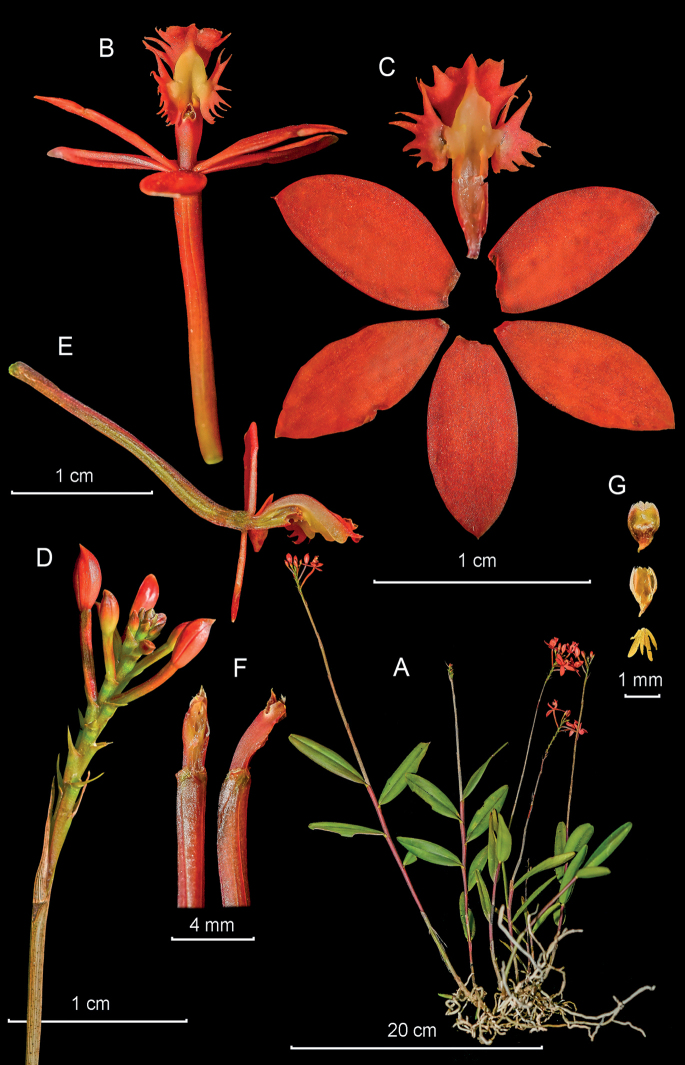
*Epidendrumparvireflexilobum* from *Arista et al. 49***A** habit **B** flower **C** dissected perianth **D** inflorescence **E** longitudinal section of flower **F** column and ovary apex from below (left) and side (right) **G** anther from above (up) and below (down), and pollinarium (Photographs by J. D. Edquén; plate prepared by A. Cisneros).

#### Additional specimens examined.

**Peru. Amazonas**: Prov. Bongará: Yambrasbamba: Perla del Imaza, La Perla Vieja, Pampa del Burro, 1871 m, 22 Aug. 2021, *Arista et al. 25* (KUELAP!); **Piura**: Huancabamba, 1 Oct. 1990, *Castillo 5* (AMO!); **San Martín**: Rioja: Pardo Miguel Naranjos, Mirador Venceremos, 1867 m, 6 Dec. 2015, *Edquén 1979* (HURP!; color plate published in Hágsater and Santiago 2020b: t. 1835, as *E.reflexilobum*).

#### Other records.

**Peru. Amazonas**: Chachapoyas: Balsas–Leimebamba, Cordillera Calla Calla, ca. 2400 m, 4 Feb. 2022, *Deza s.n.*, digital images (AMO!); *ibid. loc.* 2363 m, *Harding s.n.*, digital images (AMO!); **San Martín**: Rioja: Nuevo Cajamarca, Sector Yuracyacu, Río Yuracyacu, 1905 m, 15 Jul. 2018, *Edquén 1815*, digital images (AMO!).

#### Distribution.

Known presently from northern Peru in the Regions of Amazonas and San Martín. Growing at 1850–1905 m elevation.

#### Habitat and ecology.

Epiphytic and terrestrial in humid montane forest, secondary forest and abandoned coffee plantations, on *Coffeaarabica* L. and Citrus×aurantium L.

#### Phenology.

Flowering from July to December.

#### Taxonomic notes.

*Epidendrumparvireflexilobum* belongs to the Schistochilum Group, Secundum Subgroup, which is characterized by the caespitose habit, the erect, simple, cane-like stems, the normally elongate peduncle of the inflorescence, the erect raceme of generally non-resupinate, yellow, orange, red or purple flowers, and the lip adorned by a complex callus. The novelty is recognized by the shorter plants, up to 22–46 cm including the inflorescence, thus being one of the “dwarf species” in the *Secundum* Subgroup, as most species can be over 150 cm tall; the floral segments are short and proportionately wide, the lateral lobes of the lip are flat and not twisted and the callus is formed by a single platform with 3 short ribs, that only reaches the middle of the mid-lobe. *Epidendrumreflexilobum* occurs in the regions of Huánuco and Junín, and is overall larger, has the same red flowers with a yellow callus, and the lateral lobes of the lip spreading, twisted 90° in natural position, narrowly obovate. The novelty is also similar to *Epidendrummacrocyphum* Kraenzl. which has pink-purple flowers with a white callus formed by 3–5 straight parallel ribs on the mid-lobe, has the mid-rib longer nearly reaching the apical sinus, with two divergent bifid calli at the base of the lateral lobes, the longer segment projecting on the junction of the mid-lobe with the lateral lobes, and the lip in natural position has the mid-lobe flat, extended, the lateral lobes erect, embracing the entire column with outer margin strongly revolute. In a previous publication of *Epidendrumreflexilobum* (Hágsater and Santiago 2020a: t. 1835), the description is a mixture of the new species and *Epidendrumreflexilobum*; the latter species corresponds to the photograph on the text page, but the color plate corresponds to the new entity here described.

#### Etymology.

From the Latin *parvi*-, small, and *reflexilobum*, reflexed lobes, in reference to the smaller flowers and lobes of the lip relative to those of closely allied *E.reflexilobum*.

### ﻿ Epidendrum pomacochense

Taxon classificationPlantaeAsparagalesOrchidaceae

Hágsater, Icon. Orchid. 3: t. 374. 1999.

898449EA-20A5-5D7C-A515-E2622CF333DC

Fig. 13

#### Type material.

**Peru. San Martín**: Mirador, between Moyobamba and Pomacochas, just before Pomacochas, 1700 m, pressed 23 Nov. 1993, *hort. J. & L. Orchids sub E. Hágsater 11391* (holotype: AMO!).

#### Description.

Epiphytic or lithophytic, monopodial, branching, erect ***herb***, 15–20 cm tall. ***Roots*** 1–2 mm in diameter, basal from primary stem, fleshy, thin. ***Stems*** 12–18 × 0.15–0.4 cm, cane-like, terete, branching, branches produced from sub-apical internodes of previous stems, progressively shorter. ***Leaves*** 3–13 per stem, distributed along apical half of primary stem, and aggregate toward apex in shorter branches, distichous, articulate, sub-coriaceous, green, concolor; sheathes 0.6–1.2 cm long, tubular, striated, red; blade 1.2–4 × 0.3–0.4 cm, linear-lanceolate, apex unequally bilobed, margin entire, spreading. ***Spathe*** lacking. ***Inflorescence*** to 3 cm long including flowers, apical, racemose, arching-nutant, densely few-flowered; peduncle 6–8 mm long, terete, thin, scarcely verrucose. ***Floral bracts*** 2.0–2.5 mm long, much shorter than ovary, narrowly triangular, embracing. ***Flowers*** up to 9, resupinate, successive, 2–3 open simultaneously, sepals and petals copper-red, lip dark brick-red, with disc and column yellow, anther yellow slightly tinged brick-red on sides; fragrance none registered. ***Sepals*** partly spreading, free, membranaceous, dorsally scarcely verrucose, not carinate, 3-veined, somewhat concave, margins entire, spreading; dorsal sepal 4.6–5.5 × 3.1–4.3 mm, widely elliptic, apex rounded-obtuse; lateral sepals 5.5–7.0 × 3.8–5.5 mm, obliquely ovate, apex obtuse. ***Petals*** 4.2–6 × 1–1.5 mm, partly spreading, free, membranaceous, linear-lanceolate, obtuse, 3-veined, margin entire, spreading. ***Lip*** 7–8.4 × 6.7–9 mm, united to column, shallowly 3-lobed, transversely elliptic in outline, concave, base cuneate; ecallose, disc 5-ribed, smooth, parallel, 3 mid-ribs reaching middle of lip, lateral pair shorter; lateral lobes semi-orbicular, mid-lobe smaller, formed by two small semi-orbicular lobes, emarginate. ***Column*** 4 mm long, slightly arched with apex recurved upward, thick, apical aperture wide, triangular. ***Clinandrium hood*** short, margin entire. ***Anther*** 4-celled, obreniform, papillose. ***Pollinia*** 4, obovoid, laterally compressed, subequal, cream-colored; caudicles soft and granulose, as long as pollinia. ***Rostellum*** subapical, slit; viscarium semi-liquid. ***Lateral lobes of stigma*** small. ***Cuniculus*** a very wide cavity formed by column and lip, triangular, reaching perianth, unornamented, with a longitudinal widened slit ventrally at base of column. ***Ovary*** 6–9 mm long, terete, thin, not inflated, furrowed and scarcely verrucose. ***Capsule*** not seen.

**Figure 13.**
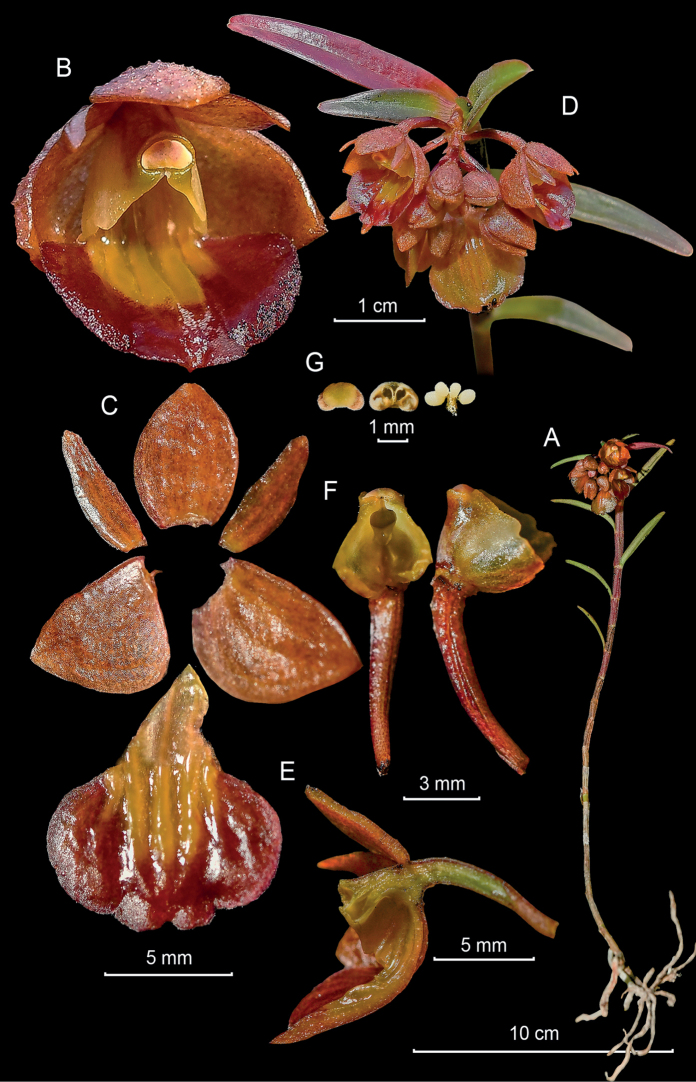
*Epidendrumpomacochense* from *Arista et al. 38***A** habit **B** flower **C** dissected perianth **D** inflorescence at apex of stem **E** longitudinal section of flower **F** column and ovary-pedicel from below (left) and side (right) **G** anther from above (left) and below (right), and pollinarium (Photographs by J. D. Edquén; plate prepared by A. Cisneros).

#### Additional specimens examined.

**Peru. Amazonas**: Perla del Imaza, La Perla Vieja, 1863 m, 22 Aug. 2021, *Arista et al. 38* (KUELAP!); Perla de Imaza, Rio Rojo, 1875 m, 25 Aug. 2021, *Arista et al. 108* (KUELAP!); road Chachapoyas–Mendoza, a little past Molinopampa, 2400 m, 15 Mar. 1998, *van der Werff 15019* (SEL!); **San Martín**: Bosque de protección Alto Mayo. Sector Venceremos, 1955 m, 21 Feb. 2017, *Edquén 195* (UNACH!) Prov. Rioja, Distrito Pardo Miguel, Chisquilla, 3278 m, 11 Apr. 2019, *Edquén 1810* (UNACH!).

#### Other records.

**Peru.***Sine loc.*, *Jenny s.n.*, digital image (AMO!); **Amazonas**: Rodríguez de Mendoza, 30 Jan. 2017, *Arbilo s.n.*, digital image (AMO!); Huamantapa, R[odríguez]. de Mendoza, 9 Apr. 2015, *Fernández s.n.*, digital image (AMO!); Área de Conservación Pampa del Burro, camino a Perla del Imaza, km 14.5, 1823 m, 14 Jul. 2022, *Hágsater 16407*, digital image (AMO!); *ibid. loc.*, km 16, 1837 m, 15 Aug. 2022, *Hágsater 16428*, digital image (AMO!); **Cajamarca**: Prov. Cajabamba, Parubamba, 2600 m, 10 Dec. 2005, *Trujillo 243*, illustrated by *M. Alcántara 1558*, copy of illustration (AMO!); **San Martín**: Prov. Rioja, Distr. Pardo Miguel Naranjos, Venceremos, 1699 m, 21 Feb. 2017, *Edquén 2025*, digital images (AMO!); *ibid. loc.*, *Edquén 2026*, digital images (AMO!); *ibid. loc.*, *Edquén 2027*, digital images (AMO!); *ibid. loc.*, *Edquén 2028*, digital images (AMO!).

#### Distribution.

Known only from northeastern Peru, in Amazonas, San Martin and Cajamarca where it is common in protected areas with wet Andean forests, at 1700–2000 m.

#### Habitat an ecology.

Epiphytic in humid montane forest, sclerophyllous shrubs, and very wet dwarf forest, and lithophytic on road-side banks of white sand and sandstone.

#### Phenology.

Flowering from January to August.

#### Taxonomic notes.

*Epidendrumpomacochense* belongs to the Diothonea Group and Subgroup, which is characterized by the monopodial, branching plants, the linear lanceolate to oblong, bilobed leaves, the racemose, arching-nutant inflorescence, the membranaceous flowers (rarely fleshy), the entire to 3-lobed, ecallose lip with the margin erose without or with 1–10 thin, smooth to erose keels, the column completely to obliquely united to the lip, and the anther reniform. *Epidendrumpomacochense* is recognized by the small plants, 15–20 cm tall, the copper-red flowers with the disc of the lip and column yellow, the sepals 4.6–7 mm long, dorsally sparsely verrucose, the column short and forming a large triangular aperture with the lip, the lip 3-lobed, transversely elliptic, the disc of the lip 5-ribbed, the ribs low, rounded, the 3 central ribs reaching the middle of the lip, the lateral lobes semi-orbicular, the mid-lobe smaller, formed by two small semi-orbicular lobes, emarginate. *Epidendrumcochabambanum* Dodson & Vásquez has larger plants up to 30 cm tall, linear leaves to 4.5 cm long, the inflorescence up to 4 cm long with flowers white tinged pink, and the lip 5-ribbed, the ribs high and apically truncate. *Epidendrumstenophyllum* Hágsater & Dodson is florally similar but the plants are up to 55 cm tall, with linear leaves, nearly acicular, 2–5.5 × 0.2–0.3 cm, the sepals up to 10 mm long.

### ﻿ Epidendrum rosulatum

Taxon classificationPlantaeAsparagalesOrchidaceae

Hágsater, E.Santiago, J.P.Arista & Edquén sp. nov.

BDEE3234-C254-5912-A112-4D39B0561CE7

urn:lsid:ipni.org:names:77320361-1

Fig. 14.

#### Type material.

**Peru. Amazonas**: Prov. Bongará, Distr. Yambrasbamba, camino a la Perla del Imaza, 1842 m, 15 Jul. 2022, *J. P. Arista*, *J. D. Edquén*, *E. Hágsater*, *E. Santiago*, *G. A. Salazar*, *E. Yrigoín*, *L. I. Cabrera & K. Edquen 278* (holotype: KUELAP!).

#### Diagnosis.

Similar to *Epidendrumcroceoserpens* Hágsater & Salas-Guerr. but the plants much smaller, up to 2.5 cm tall (vs. plants 9 cm tall), the leaves 0.8–1.2 cm long, orbicular, 4–7 forming a rosette (vs. leaves 3.5–5.6 cm long, 1–3, lanceolate), the flowers 7, ochre–yellow, with sparse red dots (vs. flowers 3–6, orange, turning pink with age), and the lip ovate-triangular, acute (vs. lip widely cordiform, base sub-cordate, apex short apiculate, margin erose-crenulate).

#### Description.

Epiphytic, sympodial, caespitose ***herb*** 1.8–2.5 cm tall, forming a small mat. ***Roots*** 2 mm in diameter, basal, fleshy, thin, white. ***Stems*** 0.6–1.0 × 0.8 cm, thickened, forming a pseudobulb, globose, compact, homoblastic, medium green, somewhat coppery, completely covered by several sheaths 5–6 mm long, non-foliar, somewhat striated when dry, light brown. ***Leaves*** 4–6, forming a rosette at apex of pseudobulb, sessile; leaves 0.8–1.2 × 0.65–0.9 cm, orbicular, apex rounded, minutely apiculate, apical margin denticulate, spreading, fleshy thickened, succulent, surface strongly rugose, adaxially dark green, margin red-brown, abaxially red-purple, margins brownish black. ***Spathe*** lacking. ***Inflorescence*** apical, sessile, racemose, densely few-flowered, not surpassing leaves. ***Floral bracts*** 4 mm long, prominent, somewhat longer than ovary, widely triangular, acute, embracing. ***Flowers*** up to 7, simultaneous, upright, ochre-yellow with sparse red dots; fragrance not detected. ***Sepals*** partly spreading, free, 3-veined, acute, margins entire, spreading; dorsal sepal 3.5–4 × 1.6–1.7 mm, narrowly triangular; lateral sepals 5.0–5.8 × 2.4–2.5 mm, ovate triangular, oblique, apiculate. ***Petals*** 3.0–3.2 × 1 mm, free, partly spreading, oblong-lanceolate, 1-veined, obtuse, margin entire, spreading. ***Lip*** 3.0–3.2 × 3.0–3.1 mm, totally united to column, entire, ovate-triangular, acute, base truncate, margins entire, spreading: ecallose and without ribs on disc. ***Column*** 2.2 mm long, thick, slightly arched, forming a 120° angle with apex of ovary. ***Clinandrium hood*** short, margin erose. ***Anther*** sub-reniform, base and apex truncate, 4-celled, cream colored with center brown and rugose. ***Pollinia*** 4, lenticular; caudicles soft and granulose, longer than pollinia. ***Rostellum*** apical, slit; viscarium semi-liquid. ***Lateral lobes of stigma*** large, about half length of stigmatic cavity. ***Cuniculus*** shallow, without penetrating ovary, very wide in column, unornamented. ***Ovary*** 3.0–3.5 mm long including pedicel, terete, not inflated, furrowed. ***Capsule*** not seen.

**Figure 14.**
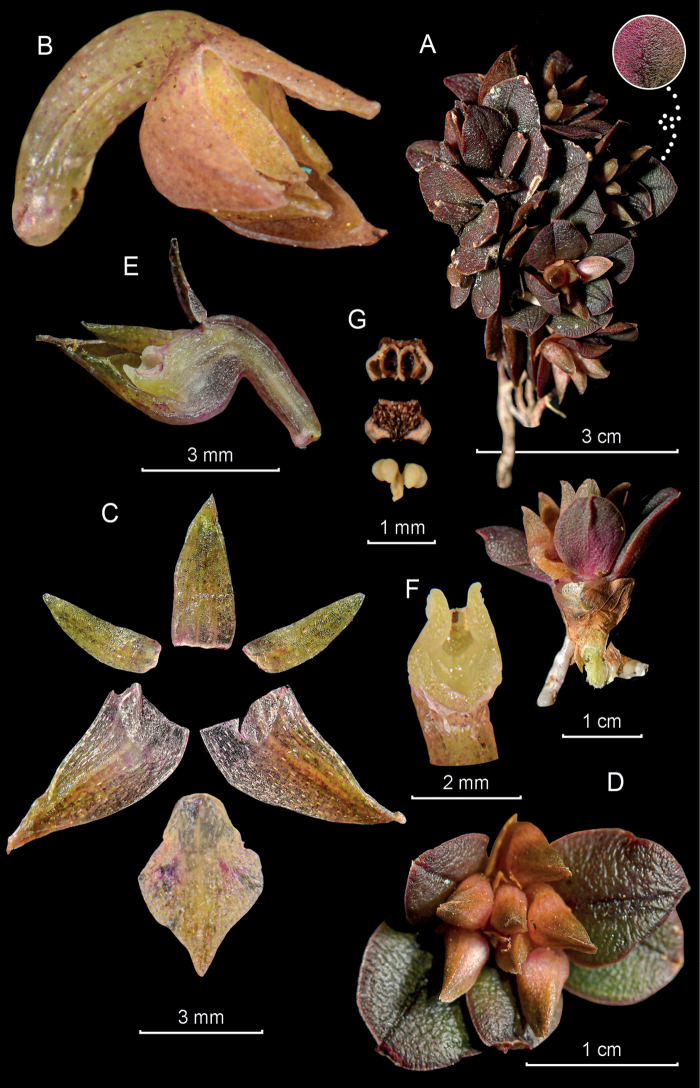
*Epidendrumrosulatum* from *Arista et al. 278***A** habit; the inset shows a slightly enlarged portion of the leaf underside **B** flower **C** dissected perianth **D** top view of inflorescence and leaves **E** longitudinal section of flower **F** column and ovary apex from below **G** anther from below (up) and above (down), and pollinarium (Photographs by J. D. Edquén; plate prepared by X. Alcántara).

#### Distribution.

Presently known only from the type. Peru, Amazonas: Prov. Bongará, Distr. Yambrasbamba, camino a la Perla del Imaza, at 1842 m.

#### Habitat and ecology.

Epiphytic in an isolated *Inga* sp. tree amidst a pasture for cattle grazing, near remnants of wet montane forest. In the crown of the tree with several individuals on branches below the leaves of the tree.

#### Phenology.

Flowering in July–August.

#### Taxonomic notes.

*Epidendrumrosulatum* belongs to the Kaloptenix Group, Serpens Subgroup, characterized by the aggregate, globose pseudobulbs with fleshy-coriaceous leaves, and a sessile inflorescence, and one or few fleshy, compact, stellate flowers, often burgundy red in color, and the lip entire, more or less cordiform. The new species is recognized for having very small plants, up to 2.5 cm tall, globose pseudobulbs, 4–6 apical, leaves forming a rosette around the inflorescence, the leaves orbicular tinged purple red with the margins brown black, the inflorescence of up to 7 flowers, ochre with some red dots, lateral sepals 5.0–5.8 mm long and the lip 3.0–3.2 × 3.0–3.1 mm, ovate-triangular, acute. *Epidendrumcroceoserpens* has 1–3 leaves per pseudobulb, the leaves lanceolate, to 5.1 cm long, green above, underside vinaceous, margin minutely erose; flowers 3–6, facing upwards, orange, turning somewhat pink with age, petals 4.2 mm long, ovate, and lip with a low wide mid-rib. *Epidendrumcitroserpens* Hágsater, Cisneros & J.Duarte has 2 leaves per pseudobulb, a 2–3 flowered inflorescence, the flowers yellowish green, a cordiform lip, short apiculate, the disc with a thick mid-rib, and a column about 5 mm long. *Epidendrumbreviyacuriense* Hágsater, H.Medina & J.Duarte has a single apical leaf, ovate, 4 simultaneous, reddish violet flowers, petals 5.0 × 1.5 mm, margin somewhat erose, the lip widely cordiform, margin irregularly dentate-erose, and disc with a thin mid-rib from the base to the middle of the disc.

#### Etymology.

From the Botanical Latin *rosulatus*, rosette (a circular cluster of leaves) shaped, in reference to the distinctive rosette formed by the leaves, which is a rare trait in the genus.

### ﻿ Epidendrum tridens

Taxon classificationPlantaeAsparagalesOrchidaceae

Poepp. & Endl., Nov. Gen. & Sp. Pl. (Poeppig & Endlicher) 2: 2. t. 103. 1838.

54C582F6-DDB3-5CB1-A333-A9A0F95DECAF

Fig. 15

#### Type material.

**Peru. [Huánuco**:] Subandin. supra arbores, Cuchero, *E. F. Poeppig s.n.* (holotype: W-R!; isotype: W-R 42400!).

#### Taxonomic synonym.

*Epidendrumtunguraguae* Schltr. Repert. Spec. Nov. Regni Veg. Beih. 8: 87 (1921). Type: Ecuador. Tunguragua: In rupibus in convalle subandina montis Tunguragua, c. 1800 m, Jun. 1886, *L. A. Sodiro 69a* (holotype: B, destroyed; illustration AMES36134!; neotype, designated by Sánchez Saldaña and Hágsater in Hágsater and Sánchez Saldaña 2015, t. 1565: Wulkan Tunguragua, 1500–2000 m, blooms in June and July, *F. C. Lehmann 6719*, K! (pencil illustration of live plant in flower, *Lehm. Ic. Pl. Tabul. 438*, K!; isoneotype AMES!).

#### Description.

Epiphytic, lithophytic or terrestrial, sympodial, caespitose, erect ***herb*** 26–100 cm tall, including inflorescence. ***Roots*** 3–4 mm in diameter, basal, fleshy. ***Stems*** 18–56 × 0.6–1.5 cm, simple, cane-like, laterally compressed toward apex, straight, green, sometimes tinged purple. ***Leaves*** 4–8, distributed along apical ¾ of stems, erect, coriaceous; plants deep green sometimes tinged purple, especially sheaths and underside of leaves; sheath 2–6 cm long, foliaceous, laterally compressed, ancipitose; blade 8.0–15 × 2–5 cm, unequal, progressively larger toward apex of stem, narrowly elliptic, 3–5 times longer than wide, apically unequally bilobed, minutely mucronate, venation and dorsal keel evident, dark green, occasionally tinged purple. ***Spathe*** lacking. ***Inflorescence*** apical, racemose, becoming pluri-racemose, producing one flower at a time, over several years from same stem; peduncle 3–4 mm long, reduced, rachis 5–7[10] mm long. ***Floral bracts*** 7–11 × 5–6 mm, much shorter than ovary, triangular, acuminate, amplexicaul. ***Flowers*** successive, one at a time from each raceme, resupinate, sepals and petals green to yellow, occasionally tinged purple, lip and column white; scented at night. ***Sepals*** 42–73 × 5–8 mm, spreading, linear-lanceolate, acuminate, 10-veined, with numerous secondary veins, margin entire, revolute. ***Petals*** 40–70 × 2.5–3.0 mm, partly spreading, linear-lanceolate, acuminate, 5-veined, with numerous secondary veins, margins entire, spreading. ***Lip*** 27–53 × 24–30 mm, united to column, 3-lobed, margin entire, spreading; bicallose, calli laminar, prominent; lateral lobes 14–26 × 6–9 mm, semi-ovate, rounded; mid-lobe 20–37 × 2–3 mm, ensiform, acute, margin entire. ***Column*** 21–25 mm long, straight to slightly arched, strongly dilated toward apex. ***Clinandrium hood*** slightly surpassing body of column, generally somewhat dentate, occasionally deeply dentate. ***Anther*** obovoid, 4-celled. ***Pollinia*** 4, semi-obovoid, laterally compressed; caudicles soft and granulose, about as long as pollinia. ***Rostellum*** apical, slit; viscarium semi-liquid, transparent. ***Lateral lobes of stigma*** reduced. ***Cuniculus*** penetrating nearly half length of ovary, unornamented. ***Ovary*** 60–100[120] × 2.0–3.5 [5] mm long including pedicel, shorter to slightly longer than apical leaf, terete, inflated, unornamented. ***Capsule*** ellipsoid, slender; pedicel 25–35 × 2.0–2.5 mm, body 45–60 × 17–21 mm, at center of capsule; apical neck 13–16 × 2.3–3.5 mm.

**Figure 15.**
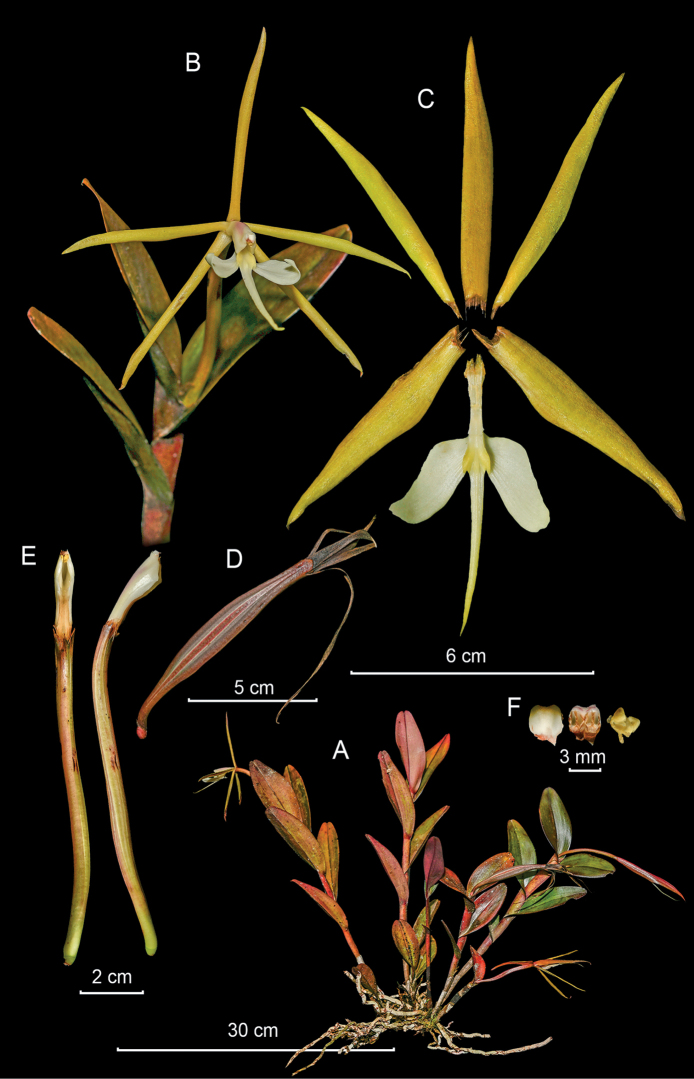
*Epidendrumtridens* from *Arista et al. 9***A** habit **B** flower **C** dissected perianth **D** developing capsule **E** column and ovary-pedicel from below (left) and side (right) **F** anther from above (left) and below (right), and pollinarium (Photographs by J. D. Edquén; plate prepared by A. Cisneros).

#### Additional specimens examined.

**Peru.***Sine loc.*, *Bennett 57* (MOL!); *Ibid. loc. Bennett 3669* (MOL!); *ibid. loc.*, 1803, *Ruiz & Pavón s.n.* (MA!); *Sine loc.*, 1876–1877, *Vidal-Sénège 68* (P!); **Amazonas**: Prov. Bongará, Dist. Yambrasbamba, Perla del Imaza, La Perla Vieja, *Arista et al. 9* (KUELAP!); **Cajamarca** Bongará near Pedro Ruiz, 2200 m, Aug. 1979, *Königer 24/1* (SEL!); Bongará, 1 Sep. 1985, *Moore s.n.* (SEL!); Bongará, Dist., Yambrasbamba, viaje al puente Vilcaniza, 1840–2020 m, 9 Jul. 1999, *Sánchez-Vega 10028* (AMO! F!); **Ayacucho**: Aina, between Huanta and Río Apurimac, 750–1000 m, 7 May 1929, *Killip 23169* (US!); **Cajamarca**: San Ignacio Huarango, Nuevo Mundo, 1140 m, 11 Mar. 2000, *Calatayud 425* (CUZ!); San José de la Alianza, Rinconada, 2200 m, 18 Mar. 2009, *Vásquez 242* (MOL!) **Cusco**: Paucartambo, Mirador, Unión-Cosñipata, 1750 m, Feb. 1994, *Moscoso 760* (CUZ!) Paucartambo, San Pedro–Cosñipata, 1480 m, Mar. 1994, *Moscoso 1112* (CUZ!); *ibid. loc.*, Mar. 1994, *Moscoso 1113* (CUZ!); Quispicanchis, Río Arazá, between Pan de Azúcar and Quince Mil Airport. 292 km from Cusco, 643 m, 10 Aug. 1991, *Nuñez 14065* (MO!); Marcapata, Murayaca, Quispicanchis, 1685 m, 6 Nov. 2006, *Villafuerte 620* (CUZ!); **Huánuco**: Leoncio Prado, La Alcantarilla, Tingo María, 650 m, 6 Jul. 1984, *Fernández 385* (USM!); Carpish, entre Huánuco y Tingo María, 2800–2900 m, 6 Feb. 1950, *Ferreyra 6713* (AMES! UC! USM!); Carpish pass, Tingo María side, 2000 m, 18 Jul. 1964, *Hutchison 5977* (UC! US!); Carpish Hill, between Huánuco and Tingo María, 2100 m, 10 Aug. 1980, *Luer 5351* (SEL!); Carpish, km 453, 2500 m, 7 May 1976, *Plowman 6070* (AMES!); San Pedro de Carpish, Mirador, *Ridoutt s.n.* (USM!); Chinchao–Carpish, 2400 m, *Woytkowski 5024* (MO! AMES!); **Junín**: Chanchamayo, La Merced, Quebrada del Carmen, 850 m, 6 May 1984, *Fernández 305* (USM!); Chanchamayo, El Refugio, San Ramón, 800 m, 16 May 1984, *Fernández 318* (USM!); Tarma, Agua Dulce, 1900 m, 16 Mar. 1948, *Woytkowski 35478* (MO!); **Loreto**: Coronel Portillo, Divisoria, entre Tingo María y Pucallpa, 1500–1600 m, 28 Feb. 1947, *Ferreyra 1677* (USM!); **Pasco**: Chontabamba, La Suiza Nueva, 2190–2200 m, 7 Jan. 2005, *Arias 70* (HOXA! MO!); Oxapampa, Sector San Alberto, P[arque]. N[acional]. Yanachaga–Chemillén, 2100 m, 18 Mar. 2005, *Ortiz 507* (HOXA! MO!); Oxapampa, Huancabamba, localidad Grapanazu, Sector San Daniel, P[arque]. N[acional]. Yanachaga–Chemillén, 2236 m, 10 Jul. 2004, *Perea 1476* (HOXA! MO!); Oxapampa, Chontabamba valley, 23 km W of Oxapampa 1900 m, 26 Jan. 1984, *Smith 5881* (MO! USM!); Huancabamba, Parque Nacional Yanachaga–Chemillén, Sector Quebrada Yanachaga. 1700–2265 m, 17 Feb. 2004, *Vásquez 29534* (HOXA! USM!); **Puno**: Carabaya, San Gaban, alrededores de San Gaban, 1810 m, 9 Mar. 2017, *Trinidad 4134* (USM!); alrededores Sandia, 2250 m, 5 Feb. 1964, *Vargas 15149* (AMES! CUZ!); **San Martín**: Cordillera Azul, Coronel Portillo, Tingo María on highway to Pucallpa, near Divisoria, ca. 1600 m, 17 Nov. 1949, *Allard 21786* (AMES! US!); **Ucayali**: Padre Abad, Parque Nacional Cordillera Azul, Divisoria, entre Tingo María y Pucallpa, 1500 m, 28 Feb. 1947, *Ferreyra 1677* (USM!).

#### Other records.

**Peru.***Sine loc.*, illustration by I. Pulgar, *Ruíz and Pavón 1282* (MA!); **Jaén**: *Ocupa-Horna s.n.*, color plate (AMO!); **San Martín**: Moyobamba above Naranjo at km 468 along Olmos–Moyobamba road, 1020 m, 9 Dec. 1990, *Bennett 4800*, illustration in Icon. Orchid. Peruviarum t. 51 (Bennett and Christenson 1993); Bosque de Protección Alto Mayo, Jan. 2015, *Collantes s.n.*, digital images (AMO!); Rioja: Pardo Miguel Naranjos, Venceremos, 1887 m, 6 Feb. 2017, *Edquén 1088*, digital images (AMO!); *ibid. loc.*, 1750 m, 26 May 2022, *Edquén 6038*, digital images (AMO!).

#### Distribution.

Widespread on the Amazonian slope of the eastern Andean range in Colombia, Ecuador, and Peru, and further northeastern into the Guiana Shield in Venezuela, at 640–2900 m.

#### Habitat and ecology.

Usually growing as a terrestrial or lithophytic on roadside embankments and sometimes epiphytic in montane wet forest and dwarf forest on white sand.

#### Phenology.

Flowering throughout the year, fruiting mainly from June to September.

#### Taxonomic notes.

*Epidendrumtridens* belongs to the Nocturnum Group which is characterized by the sympodial, caespitose plants, cane-like, non-fusiform stems, successive flowers on a short, racemose or pluri-racemose inflorescence, without spathaceous bracts, and large, star-shaped flowers, with similar sepals and petals; the flowers are mostly indistinguishable in shape. The species is recognized by the dark green plants, the underside of the leaves and sheaths tinged with purple, laterally compressed stems, 4–8 erect leaves, generally longer toward the apex of the stem, length/width 3:1–5:1 (8.5–15 × 2–5 cm), distributed along the apical ¾ of the stems, green often tinged with purple; the ovary 60–100 [120] mm long, equal or occasionally longer than apical leaf, the sepals 42–73 mm long, the lateral lobes of lip semi-ovate, rounded to acute, acuminate, 14–26 mm long; the mid-lobe 20–37 mm long, the column 21–25 mm long; body of the capsule centered. *Epidendrumnocturnum* Jacq. is widely distributed from Florida to Bolivia, has green plants, terete stems, smaller leaves distributed along the apical 2/3 of the stems, a short ovary, 50–70 mm long, and the body of the capsule occupying nearly its whole length. *Epidendrumtumuc-humaciense* (Veyret) Carnevali & G.A.Romero is found along the Guiana Shield, and at lower altitudes in the Amazon basin in Colombia, Venezuela, Guyana, Surinam, French Guiana and the northern border of Brazil. Plants are frequently vinaceous, or yellow-green, it has numerous, shorter, narrower, erect leaves (3–9.2 × 1.2–2.8 cm), distributed throughout the stems, the basal ones generally longer; sepals and petals 48–81 mm long; body of the capsule occupying the apical half of the fruit.

### ﻿ Epidendrum weigendii

Taxon classificationPlantaeAsparagalesOrchidaceae

Hágsater & Cisneros, Icon. Orchid. 18(2): t. 1899. 2021.

6C43306B-17F2-56EC-B15C-8F6C0D09E48D

Fig. 16

#### Type material.

**Peru. Amazonas**: Chachapoyas: Molinopampa: Road Chachapoyas to Mendoza, km 36, cloud forest, on sandstone, 2800 m, 5 June 1998, *M. Weigend*, *T. Franke*, *J. Skrabal & M. A. González 98/429* (holotype: F-2211499!; isotype: USM!).

#### Description.

Epiphytic, sympodial, scandent, erect ***herb*** up to 58 cm tall, new stems produced from a middle internode of previous stem. ***Roots*** fleshy, from base of primary stem or occasionally from base of upper stems. ***Stems*** 8–15 × 0.3–0.5 cm, erect, simple, cane-like, terete, basal 3/4 of stem covered with non-foliar sheaths. ***Leaves*** 2–3, aggregate toward apex of stem, spreading, alternate; sheaths 0.6–2.5 × 0.3–0.5 cm, tubular, striated; blades 2.1–9.6 × 0.9–2.1 cm, unequal in size, elliptic-oblong, acute, sub-coriaceous, medium green. ***Spathe*** lacking. ***Inflorescence*** 4–6 cm long, apical, from mature stem, racemose, arcuate; peduncle 1.4–2.3 × 0.3 cm, somewhat laterally compressed, sometimes with a single bract near base, 8 mm long, acute, embracing; rachis 2.3–5 cm long, arching-nutant. ***Floral bracts*** 3–6 mm long, much shorter than ovary, decreasing in size, triangular, acute, embracing. ***Flowers*** 7–11, opening in succession, until most open at same time, resupinate, facing downward, medium green, lip pale green, apex of column around anther slightly tinged purple, anther green; fragrance none. ***Sepals*** free, spreading, fleshy, 5-veined, margins entire, spreading; dorsal sepal 13–16 × 5.4–6.0 mm, narrowly obovate, obtuse; lateral sepals 14.5–15.0 × 5.4–6.0 mm, obliquely oblong oblanceolate, somewhat falcate, acute. ***Petals*** 13.5–16 × 3.5–6.0 mm, free, spreading, oblanceolate, apex sub-acute to rounded, 3-veined, margin entire, spreading. ***Lip*** 14.8 × 17.7 mm, united to column, deeply 3-lobed, fleshy, wider than long, base strongly cordate, apex emarginate, margin entire, spreading; bicallose, calli globose, slightly separate, conspicuous, disc with three parallel, thick, low ribs running down middle, lateral ribs arching closer to mid-rib at apex, reaching apical sinus of lip; lateral lobes 7.0 × 8.0–9.0 mm, semi-orbicular; mid-lobe 6.7 × 10.9–12.5 mm, formed by two small, semi-orbicular lobes, with a short, narrow isthmus 1.6 mm long. ***Column*** 10 mm long, somewhat thick toward apex, truncate, straight. ***Clinandrium hood*** reduced, margin entire. ***Anther*** globose, apex emarginate, apical surface minutely echinate, 4-celled. ***Pollinia*** obovoid, laterally compressed, subequal; caudicles granulose, shorter than pollinia. ***Rostellum*** apical, slit; viscarium semi-liquid. ***Lateral lobes of stigma*** not seen. ***Cuniculus*** penetrating the apical one third to one half of the pedicellate ovary. ***Ovary*** 16–20 × 3 mm, slightly inflated ventrally along apical half, thin, terete, somewhat arcuate, furrowed. ***Capsule*** [immature] 45 × 0.6 mm long, narrowly ellipsoid; pedicel 0.7 × 0.2 mm, body 25–30 × 0.6 mm.

**Figure 16.**
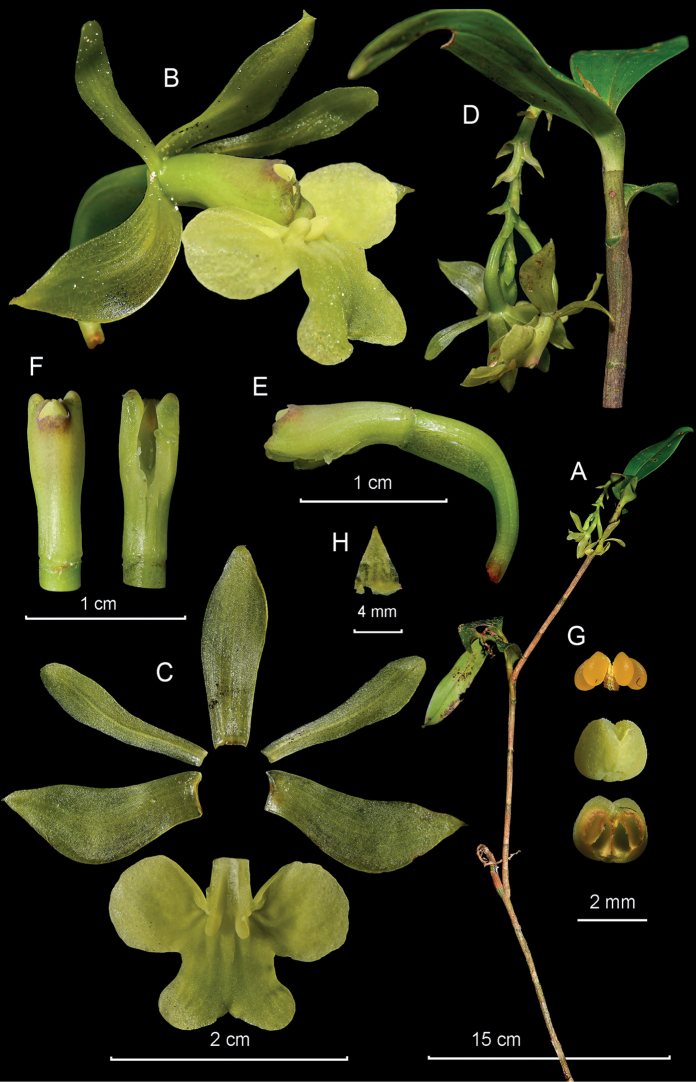
*Epidendrumweigendii* from *Arista et al. 253***A** habit **B** flower **C** dissected perianth **D** inflorescence at apex of stem **E** column with ovary-pedicel from side **F** column and ovary apex from above (left) and below (right) **G** pollinarium and anther from above (up) and below (down) **H** floral bract (Photographs by J. D. Edquén; plate prepared by X. Alcántara).

#### Additional specimens examined.

**Peru. Amazonas**: Prov. Bongará: Distr. Yambrasbamba: Pampa del Burro, Perla del Imaza, a 100 m de la carretera, en bosque chaparro esclerófilo, 1886 m, 14 Aug. 2022, *Arista et al. 253* (KUELAP!); Chachapoyas: Molinopampa: along Río Ventilla, 1–2 km W of Molinopampa, 2350–2400 m, 23 Aug. 1962, *Wurdack 1485* (US!).

#### Distribution.

Known presently from three collections from the ACPPB and from near Molinopampa, Amazonas, on the border with San Martín, northeastern Peru, 1800–2800 m.

#### Habitat and ecology.

Epiphytic in dwarf forest on white sand of Andean tepui, dwarf sclerophyllous forest and cloud forest.

#### Phenology.

Flowering from June to August.

#### Taxonomic notes.

*Epidendrumweigendii* belongs to the Incomptum Group. It is recognized by the very thin stems, 2–3 elliptic-oblong leaves aggregate at the apex, the successive flowers, until most open at same time, the flower green, the lateral lobes of the lip orbicular, as are the two lobes of the mid-lobe, the disc of the lip with 3 short parallel ribs, and the short radiating ribs at base of lateral lobes. *Epidendrumtamaense* has the floral segments strongly reflexed and a strongly arcuate ovary, sepals 15–21 mm long, lateral sepals acute to acuminate, with a conspicuous dorsal keel, petals obtrullate, and the lip larger, 22–24 × 33 mm, bicallose, with a single mid-rib.

## Supplementary Material

XML Treatment for Epidendrum acrobatesii

XML Treatment for Epidendrum brachyblastum

XML Treatment for Epidendrum cryptorhachis

XML Treatment for Epidendrum echinatiantherum

XML Treatment for Epidendrum forcipatum

XML Treatment for Epidendrum imazaense

XML Treatment for Epidendrum mavrodactylon

XML Treatment for Epidendrum ochrostachyum

XML Treatment for Epidendrum olorteguii

XML Treatment for Epidendrum parvireflexilobum

XML Treatment for Epidendrum pomacochense

XML Treatment for Epidendrum rosulatum

XML Treatment for Epidendrum tridens

XML Treatment for Epidendrum weigendii

## ﻿Additional information

### Author ORCIDs

Jessy Patricia Arista https://orcid.org/0000-0001-8334-5538

Eric Hágsater https://orcid.org/0000-0002-2371-9427

Elizabeth Santiago https://orcid.org/0000-0002-1368-9025

José D. Edquén https://orcid.org/0000-0002-7289-3974

Elí Pariente https://orcid.org/0000-0002-9197-0218

Manuel Oliva https://orcid.org/0000-0002-9670-0970

Gerardo A. Salazar https://orcid.org/0000–0002–5203–5374

### Data availability

All of the data that support the findings of this study are available in the main text or Supplementary Information.
